# A Global Imprint of Gadolinium-Based Contrast Agents Used in Magnetic Resonance Imaging in Drinking Water

**DOI:** 10.1021/acs.est.6c06733

**Published:** 2026-06-30

**Authors:** Vanessa Hatje, Franciele Castro Novais, Anna Maria Orani, Maria Elisabete Machado, Pere Masque, Marc Metian

**Affiliations:** † Centro Interdisciplinar de Energia e Ambiente (CIEnAm), 28111 Universidade Federal da Bahia, Salvador, Bahia 40170-115, Brazil; ‡ Instituto de Química, Universidade Federal da Bahia, Salvador, Bahia 40170-115, Brazil; § IAEA Marine Environment Laboratories, Department of Nuclear Sciences and Applications, International Atomic Energy Agency, 98000 Monaco, Principality of Monaco; ∥ School of Natural Sciences, Centre for Marine Ecosystems Research, 2498 Edith Cowan University, Joondalup, Western Australia 6027, Australia

**Keywords:** Gadolinium-based contrast agents, Emerging contaminants, Human exposure and regulation

## Abstract

Since the 1980s, rare earth elements (REE), particularly gadolinium (Gd) used in gadolinium-based contrast agents (GdCA), have revolutionized Magnetic Resonance Imaging (MRI) diagnostics. However, the near-complete persistence of these compounds through wastewater treatment processes has led to widespread global environmental contamination. Here, we present a global assessment of REE concentrations and GdCA signatures in tap water from 148 cities across 89 countries and Antarctica, where anthropogenic Gd ranges from 0.1 to 61.2 ng L^–1^ and accounts for up to 99% of total dissolved Gd. Contamination hotspots are concentrated in Europe and North America. While Gd levels scale with MRI usage and wastewater generation, the primary driver on contamination exposure is the local drinking water source. These results reveal Gd as a sensitive global tracer of wastewater influence on drinking-water systems. Although current concentrations pose limited immediate health risks, their worldwide prevalence highlights the emerging threat of pharmaceutical pollution and the need for rapid advances in wastewater treatment, environmental monitoring, and water-resource management.

## Graphical abstract

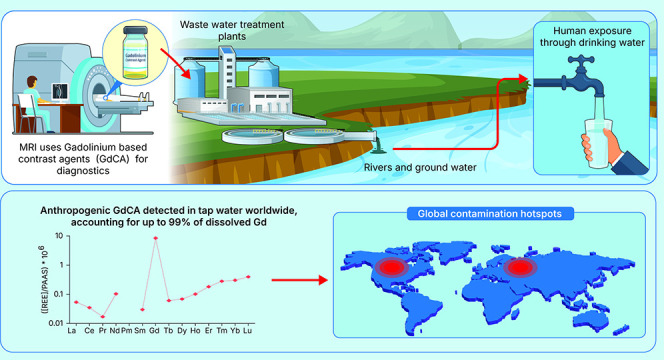

## Introduction

Water crises are intensifying worldwide (ref 1 and references therein), making water reclamation and reuse increasingly important strategies to address scarcity. These approaches offer promising solutions to alleviate scarcity, supporting applications in irrigation, industry, groundwater recharge, and even potable water supply (ref 2 and references therein). Yet, pollution remains a critical barrier to safe and sustainable water use, a threat likely to worsen under climate change.^3,4^ Its impacts span agriculture, economic growth and human health.^5,6^

Current global water indicators often fail to capture all risks associated with drinking water contamination that may be of concern to consumers.^1^ A growing area of interest involves emerging contaminants, including pharmaceuticals and technologically critical elements.^7^ Among these are the rare earth elements (REE), a group of 17 chemical elements comprising the 15 lanthanides, along with scandium and yttrium. REEs are widely distributed in the Earth’s crust and occur in various mineral sources. Some, such as La and Ce, have crustal abundances comparable to copper (Cu) and lead (Pb). Certain REEs, particularly gadolinium (Gd), have attracted attention due to their dual use: industrially, in high-technology applications such as high-performance magnets, nuclear fuel rods, and solid-state refrigeration systems, and pharmaceutically, as a contrast agent in magnetic resonance imaging (MRI) (ref 8 and references herein).

Since the late 1980s, gadolinium-based contrast agents (GdCA, also referred to as GBCA) have been widely used in MRI, leveraging Gd’s strong paramagnetic properties to enhance soft tissue contrast and improve lesion detection.^9^ To date, about ten GdCAs have been approved worldwide by national regulatory agencies. However, concerns have emerged regarding the stability and potential toxicity of GdCAs, which may release free gadolinium ions (Gd^3+^) both *in vivo* and into the environment. Free gadolinium ions (Gd^3+^) are highly toxic due to their similarity in size to calcium ions (Ca^2+^), which enables them to block voltage-gated calcium channels even at very low concentrations^10^ and references therein. To mitigate this toxicity, Gd (1.1–1.3 g Gd per scan^11^) is administered in a chelated form, either as linear or macrocyclic chelates, which significantly reduces its acute toxicity and allows for safe clinical application. Due to their chemical stability and low reactivity, these chelated complexes are almost entirely unmetabolized and are excreted rapidly, within 1.5 to 30 h after injection, depending on the complex type and the patient’s renal function, ultimately entering the sewage system.^12,13^ As most wastewater treatment plants cannot remove GdCAs,^14^ they are discharged with treated effluents into surface waters, including rivers, lakes, coastal seas,^8,15^ and can also infiltrate aquifers,^16,17^ ultimately contaminating drinking water sources that supply household tap water.^15,18–20^ The potential health impacts of human exposure to GdCAs through drinking water remain unclear,^21^ and their long-term toxicity is still poorly understood.^22,23^ However, emerging evidence suggests that GdCAs may degrade and accumulate in human tissues, including bone and brain,^24^ and have been associated with nephrogenic fibrosis.^12^ Despite their high oral bioaccessibility in humans,^23^ GdCAs remain unregulated, as is the case for all rare earth elements.

Here we provide the first global assessment of anthropogenic Gd in drinking water, spanning 148 cities across 89 countries and Antarctica (Figure 1), addressing a major gap in water quality monitoring and environmental health research. By integrating key drivers of Gd variability, such as wastewater production, MRI density, and the Human Development Index (HDI), our analysis reveals that Gd is a pervasive global contaminant and a highly sensitive tracer of wastewater intrusion into drinking water systems. Our findings underscore the global reach of Gd contamination and highlight the urgent need for systematic monitoring and management of drinking water resources to safeguard public health.

**Figure 1:**
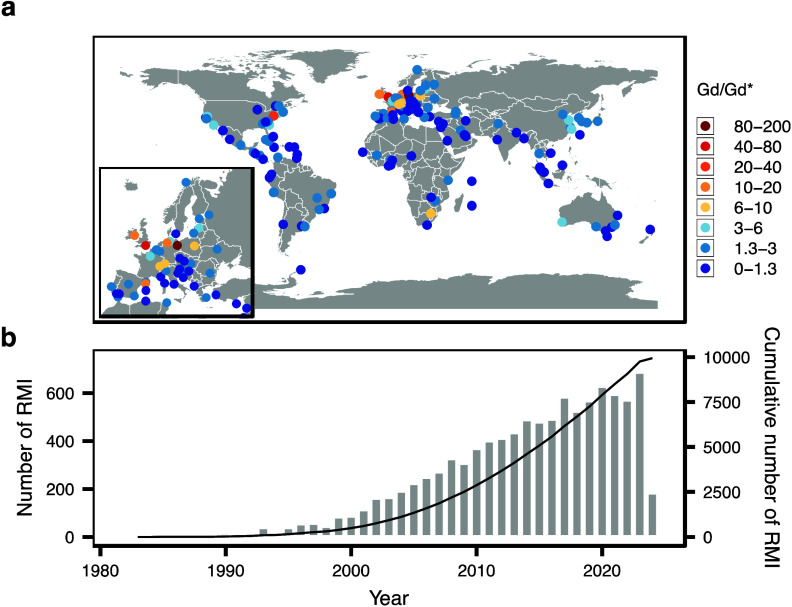
(a) Overview of global sampling locations and corresponding Gd anomalies (Gd_SN_/Gd_SN_*). Anomalies above 1.3 indicate the presence of anthropogenic Gd. (b) Number of new magnetic resonance imaging (MRI) units per million inhabitants per year and cumulative number of units over time worldwide, combining data from the Organization for Economic Cooperation and Development countries and selected non-OECD countries (OECD, 2025).

## Materials and Methods

Drinking water samples were collected from 148 cities across 89 countries, including Antarctica, between 2020 and 2026 (Figure 1a). Most cities were represented by a single sample, except Berlin (Germany), Rio de Janeiro (Brazil), and New York (USA), Lalitpur (Nepal), and London (UK) where additional samples were collected to assess small-scale variability.

In addition to samples collected by authors, we engaged scientists participating in IAEA projects and leveraged our network to collect and ship samples to the IAEA Marine Environment Laboratories and the Universidade Federal da Bahia. This approach substantially expanded spatial coverage, enabling inclusion of regions that would not have been accessible otherwise.

Given that GdCAs are regulated pharmaceuticals and the only significant anthropogenic source of Gd, and considering the low susceptibility of REE to contamination, standardized, simple protocols were applied by all collaborators. Potable water was collected directly from taps in preconditioned plastic bottles (new mineral water or acid-cleaned plastic bottles) rinsed three times with the sampled water. In some cases, domestic filters (e.g., activated carbon, UV, or sand) may have been present, but their efficiency in removing GdCAs is unknown, although expected to be minor. Because our focus was on human exposure via tap water as actually consumed, the presence of such filters does not affect the interpretation or significance of the results.

The studied sites ranged from major metropolis with extensive MRI use to smaller or low-income cities with limited healthcare infrastructure. Once in the laboratory, samples (500 mL) for total REE analysis were acidified with doubly distilled HCl (pH 1.8 ± 0.2) and stored at room temperature for up to three years prior to analysis. Selected samples exhibiting anthropogenic anomalies were tested for speciation analyses. However, not all samples showed detectable Gd species, despite showing Gd anomalies, likely due to post-collection degradation. As samples intended for speciation could not be acidified, they had to be analyzed within a few months of collection.

### REE Analysis

All samples were analyzed in duplicates at the Chemical Oceanography Laboratory, Federal University of Bahia (Brazil), following an adaptation of Hatje et al.^25^ and Novais et al.^26^ Samples were acidified with doubly distilled HCl (pH 1.8 ± 0.2), filtered through 0.22 μm nitrocellulose membranes (MF-Millipore, Merck), and irradiated for 4 h in a UV bath (∼16 mW cm^–2^, mercury lamp, UVO Cleaner Model 342, Jelight Co., Inc.). REE were preconcentrated using NOBIAS-chelate PA-1 resin (2 cm microcolumns, 27 μL, GlobalFIA). Prior to loading, the sample pH was adjusted to 4.7 ± 0.2 with NH_4_OH. Columns were rinsed with 8 mL HNO_3_ (1 mol L^–1^), conditioned with NH_4_Ac (0.05 mol L^–1^, 3 min), and loaded with 50 mL of sample at 0.4 mL min^–1^ (Dynamax peristaltic pump, Rainin). After a 3 min rinse with NH_4_Ac, analytes were eluted with 1.5 mL HNO_3_ (1 mol L^–1^) and diluted to 4.5 mL with Milli-Q water. For each batch, procedural blanks were prepared using REE-free tap water, obtained by passing tap water through two NOBIAS-chelate PA-1 resin columns connected in series and collected in precleaned 2 L LDPE bottles.

REE concentrations were determined by inductively coupled plasma mass spectrometry (ICP-MS, iCAP RQ, Thermo Scientific, Germany) with a micromist nebulizer, forward power of 1550 W, plasma gas flow of 10 L min^–1^, auxiliary gas flow of 0.8 L min^–1^, and a residence time of 0.01s. The isotopes ^89^Y, ^137^Ba, ^139^La, ^140^Ce, ^141^Pr, ^146^Nd, ^147^Sm, ^153^Eu, ^157^Gd, ^159^Tb, ^163^Dy, ^165^Ho, ^166^Er, ^169^Tm, ^172^Yb, and ^175^Lu were chosen based on natural abundances and to minimize isobaric and polyatomic interferences, which were continuously monitored. A mixed solution of Tb, Gd, and La, and another of Ce, Pr, Nd, Sm, and Ba (both 1 μg L^–1^), were analyzed every 10 measurements to track oxide formation. Oxide production was minimized by maintaining the ^140^Ce^16^O/^140^Ce ratio at 0.01 for all analyses. Potential interferences from ^137^Ba^16^O^+^ were negligible (ratios <0.1%).

All reagents were of high purity: HCl and HNO_3_ (double-distilled, Merck, Germany), NH_4_OH solution (25%, Merck), and CH_3_COOH (100%, Merck). Ultrapure water (>18.2 MΩ·cm) was obtained from an A10 Milli-Q system (Millipore, Bedford, MA, USA). REEY quantification was performed using a 21-point calibration curve (0.1–400 ng L^–1^) prepared from a multielement REEY standard solution (Y, La, Ce, Pr, Nd, Sm, Eu, Gd, Tb, Dy, Ho, Er, Tm, Yb, Lu; 10 ppm, Claritas-Spex, CertiPrep). Blanks were run after every tap water sample. Indium (In) was used as an internal standard at 20 ng L^–1^. Addition-recovery tests were carried out every 10 samples, using REE-free tap water spiked with multielement standard solutions, acidified with HCl to pH ∼ 1.8, and stored in precleaned LDPE bottles. REE-free tap water was obtained passing the water through two NOBIAS-chelate PA-1 resin columns connected in series. The resulting REE-free water was preconcentrated following the same procedure applied to the actual samples, and REE concentrations were determined by ICP-MS to confirm the absence of detectable levels.

There are no reference materials for REE (including Gd) or GdCAs in tap water. Precision and accuracy were assessed through recovery experiments. For REE, recoveries exceeded 92% (92% for Yb to 99% for Tb). For GdCA, recoveries at three spike levels (0.1, 18, and 60 ng L^–1^) were 97%, 99%, and 99%, respectively. The propagated uncertainty was determined using the Nordtest method. The expanded uncertainties ranged from 10.2% for Gd to 13.8% for Nd (k = 2). Further details on method validation are provided in Novais et al.^26^

### GdCAs Speciation Analysis

Speciation analysis was performed at the IAEA Marine Environment Laboratories in Monaco. A total of 75 samples for which particularly high Gd concentrations and/or anomalies had been detected, underwent speciation analysis. This was carried out using High Performance Liquid Chromatography (HPLC) coupled with ICP­(TQ)­MS.^100^ In brief, nonacidified samples were filtered using 0.45 μm membranes and disposable plastic syringes. The filtrates were introduced into an HPLC system equipped with a PRP X-100 anion exchange column. A gradient of aqueous mobile phases 135 mM and 150 mM ammonium nitrate (NH_4_NO_3_) was applied over a 30 min run time. This method was developed and validated for the selective detection and quantification of three commonly used GdCAs (Figure S1): linear Magnevist (Gd-DTPA), macrocyclic Dotarem (Gd-DOTA), and Prohance (Gd-DO3A) that could be obtained at the time of the method development. A table summarizing the main analytical features of the method is presented in supplementary data (Table S1).

### Numerical Procedures

REE concentrations are typically normalized to a natural reference (e.g., water or rock) to remove the Oddo–Harkins pattern, enabling easier interpretation and comparison across studies. This produces a smooth distribution that helps identify anomalies (enrichment or depletion), estimate expected natural values and assess data quality. Here, REE data were normalized to Post Archean Australian Shale (PAAS) values (Pourmand et al., 2012), and geogenic Gd (Gd*) and anomalies (Gd_SN_/Gd_SN_*) were estimated using a third-order polynomial fit excluding Ce and Eu.^27–29^ The anthropogenic contribution (Gd_AN_) was calculated as [Gd]­(measured) – [Gd*]­(calculated).

Anomalies for Ce were calculated using their normalized concentration as follows:^30^

$$\mathrm{Ce}/{\mathrm{Ce}}^{*}={\mathrm{Ce}}_{\mathrm{PAAS}}/{\left({\mathrm{La}}_{\mathrm{PAAS}}\times {\mathrm{Pr}}_{\mathrm{PAAS}}\right)}^{1/2}$$

## Results

Normalized REE concentrations (REE_SN_) showed systematic enrichment of heavy REE (HREE) relative to light REE (LREE) (Figure 2; Table S2), with Yb_SN_/Nd_SN_ ratios reaching 51 in Tokyo (Japan) and Dy_SN_/Nd_SN_ up to 5.8 in London (UK). Exceptions were limited to drinking water samples from Antarctica (lakes and tap water), Qatar, Trinidad and Tobago, and Saint Maarten. REE_SN_ patterns revealed positive anthropogenic Gd anomalies in several countries (Figure 2; Table S2), while negative Ce anomalies were common but often linked to elevated La concentrations.

**Figure 2:**
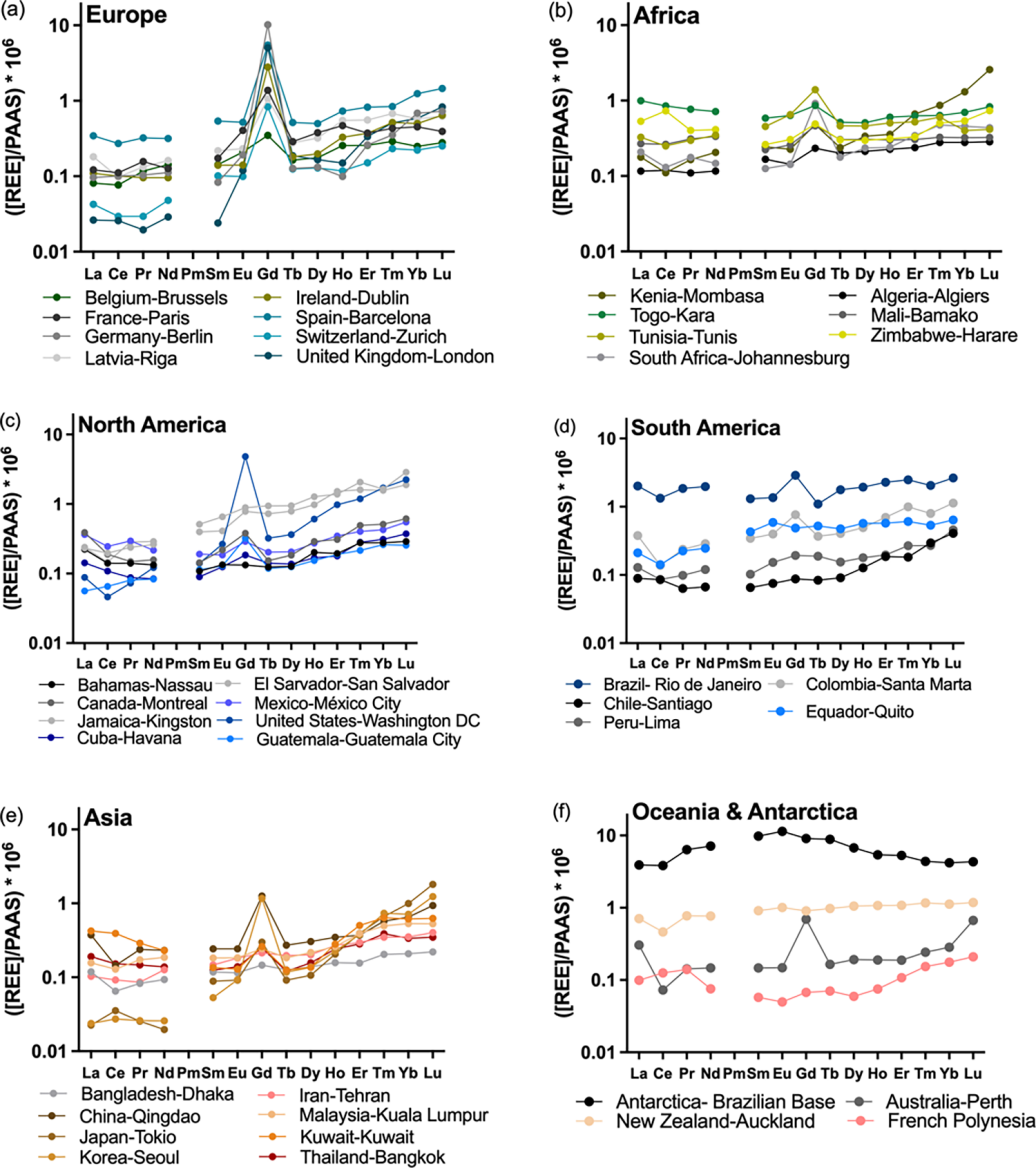
PAAS-normalized REE concentrations for selected locations worldwide across different regions. Distinct positive anthropogenic gadolinium (Gd) anomalies (observed as Gd peaks) are evident globally, indicating widespread contamination of drinking water.

Total REE concentrations (ΣREE) in drinking water varied widely between and within regions (Table S3), with a global mean of 164 ± 484 ng L^–1^ and a median of 39.5 ng L^–1^. Europe showed the lowest average ΣREE (38.7 ± 31.1 ng L^–1^; median 31.0 ng L^–1^), while Antarctica exhibited the highest (1071 ± 230 ng L^–1^; median 1063 ng L^–1^). Exceptionally high ΣREE (up to 3330 ng L^–1^) were recorded in Africa (e.g., Lomé, Togo; Benin, Nigeria; Mahé Island, Seychelles; Lusaka, Zambia) and Asia (Tangerang, Indonesia), with several drinking water samples showing visible dark coloration due to high particulate content.

Total Gd concentrations (Gd_Total_) spanned 3 orders of magnitude, from 0.35 ng L^–1^ (Monaco) to 204 ng L^–1^ (Benin, Nigeria) (Table S3). The highest values in Africa (Figure 3) were linked to geogenic sources. Excluding samples with high particulate loads, geogenic Gd ranged from 0.17 to 66.9 ng L^–1^, with maxima in Antarctica.

**Figure 3:**
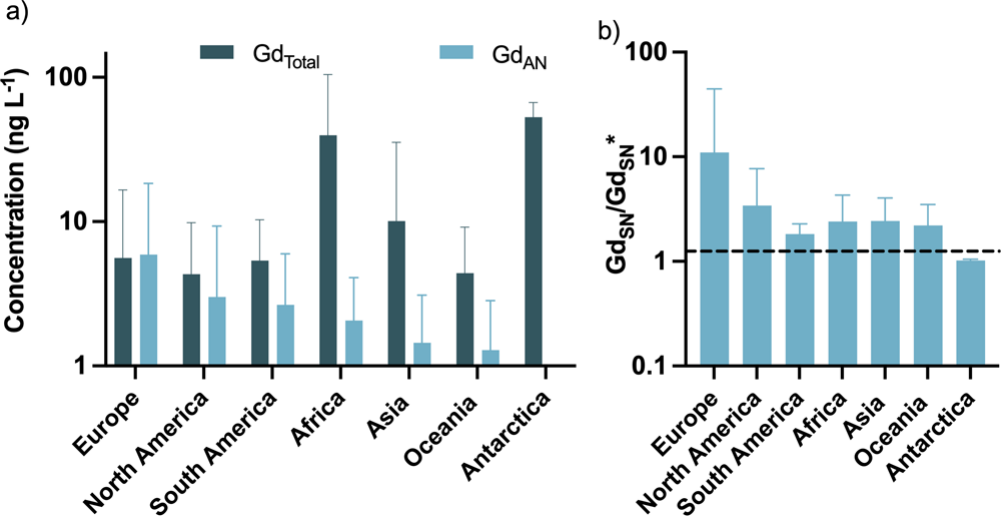
Gd concentrations and anomalies (Gd_SN_/Gd_SN_*) in drinking water. a) Regional averages (±SD) of total Gd (Gd_Total_) and anthropogenic Gd (Gd_AN_) concentrations (ng L^–1^). Only samples with Gd anomalies (Gd_SN_/Gd_SN_*) > 1.3 were included in the calculation of average Gd_AN_ concentrations. b) Regional averages (±SD) of Gd anomalies (Gd_SN_/Gd_SN_*), with the anthropogenic reference threshold (Gd_SN_/Gd_SN_* > 1.3) indicated.

Anthropogenic contributions (Gd_AN_) accounted for up to 99% of the Gd_Total_ in some instances, with the highest values observed in Europe (i.e., Berlin, Germany (61.2 ng L^–1^; 99%), London, UK (29.9 ng L^–1^; 98%), Barcelona, Spain (30.0 ng L^–1^; 91%), and Warsaw, Poland (5.99 ng L^–1^; 88%)), and in North America (Washington, DC. USA; 27.8 ng L^–1^; 95%). High anthropogenic contributions were also recorded in Johannesburg, South Africa (4.75 ng L^–1^; 86%); Tokyo, Japan (1.57 ng L^–1^; 87%); Qingdao, China (6.19 ng L^–1^; 81%); and Perth, Australia (3.07 ng L^–1^; 73%).

Anthropogenic Gd anomalies (i.e., Gd_SN_/Gd_SN_* > 1.3) occurred in 62 of the 89 countries, with highest prevalence in Europe (75%), followed by the Americas (60%), Africa (57%), Asia (53%) and Oceania (33%) (Figure 3). Anomaly values varied markedly across and within countries and cities. In Europe, Berlin (Germany) showed the largest Gd anomaly (187), followed by London (UK; 54), Dublin (Ireland; 18) and Barcelona (Spain; 11). Washington, DC (USA) recorded 21, while Gd_SN_/Gd_SN_* maxima in other regions were up to 30-fold lower.

Within country, city-level variability was also pronounced (Table S2; Figure 4) with anomalies differing by up to 60-fold in Germany, 9-fold in Spain, 4-fold in France, 11-fold in the USA, and 6-fold in Japan. Within-city variation was examined in detail only for Berlin, Germany (Figure S2). Other within-city variability data can be found in Table S2. Gd concentrations varied by up to 25-fold across different districts of Berlin and exhibited large variability in Gd_SN_/Gd_SN_* between East and West Berlin. Nevertheless, all samples from Berlin displayed Gd anomalies.

**Figure 4:**
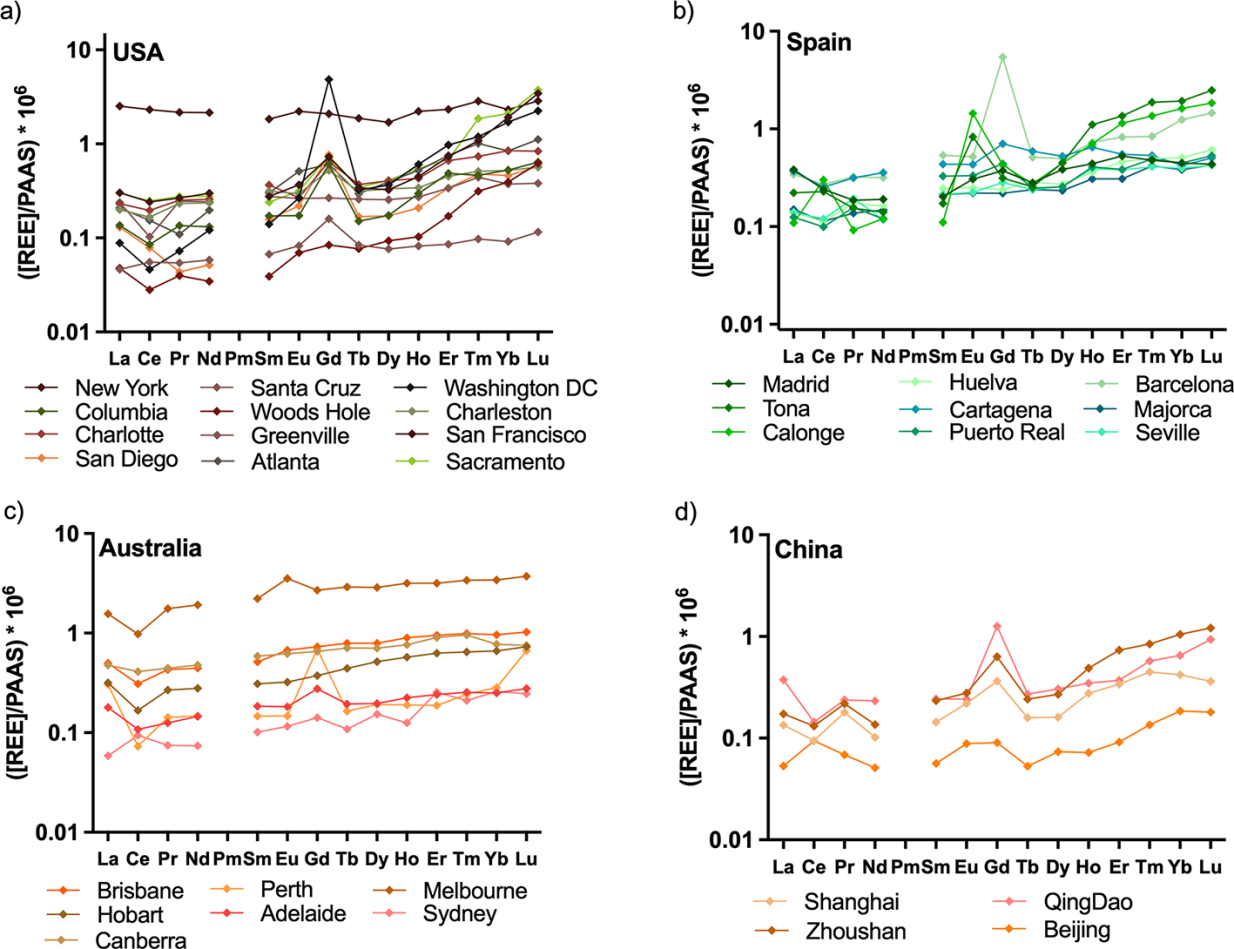
PAAS-normalized REE concentrations for selected cities in USA, Spain, Australia, and China. Substantial variability can be observed between cities in terms of Gd anomalies (Gd_SN_/Gd_SN_*, i.e., Gd peaks). Complete city-level data is presented in Table S2.

Speciation analysis of selected samples with a Gd anomaly (Table S4) showed Dotarem (Gd-DOTA) as the dominant Gd-based contrast agent, accounting for between 22 to 97% of the Gd concentrations. ProHance (Gd-HP-DO3A) and Magnevist (Gd-DTPA) were present in some samples, however, below quantification limits.

## Discussion

Potable water supplies can exhibit a wide range of compositions, whether derived from river water, groundwater, or desalination; consequently, ΣREE concentrations in tap water are highly variable at national, regional and global levels (Table S2 and S3). While the median is 39.5 ng L^–1^, samples from Benin (Nigeria), Tangerang (Indonesia), and Antarctica were up to 80-fold higher. This variability in ΣREE concentrations is primarily controlled by reactions between water and REE-bearing minerals in aquifer or source rocks during weathering, as well as interactions with clay minerals and Fe–Mn oxides/oxyhydroxides coating these and other mineral phases (Goldstein & Jacobsen, 1988b; Smedley, 1991). In addition, conditions such as pH, dissolved organic carbon (DOC), alkalinity, ionic strength, the presence of complexing ligands (e.g., carbonates and organic matter), and the amount of suspended particulate material exert important controls, as do water treatment processes including coagulation, filtration, and ozonation. As such, high ΣREE concentrations in Antarctica, for instance, do not indicate contamination, but rather reflect natural geochemical composition, in this case associated with the occurrence of REE-bearing minerals (i.e., monazite).^31^

### Global Spread of Gadolinium from MRI Diagnostics into Drinking Water

Since their introduction in the 1980s, GdCAs have become crucial to MRI (>150 million scans performed annually worldwide), with 40–50% of all examinations now involving their use. Over the same period, both the global availability of MRI units (Figure 1b), a proxy for potential GdCA usage, and the annual number of MRI examinations have increased substantially, reflecting the expansion of diagnostic imaging capacity worldwide. Among countries with available data, density of MRI units varies widely, from 0 to 53 units per million inhabitants (Table S5), highlighting global disparities in healthcare infrastructure and, consequently, variability in GdCA discharges to wastewater systems. In fact, MRI density increases sharply at HDI values between 0.5 and 0.9 but varies largely (10 to <50 units per million inhabitants) among countries with high HDI (>0.8) (Figure S3). Notably, 56% of the countries included in our study operate fewer than 10 MRI units per million inhabitants, 29% have between 10 and 20 units per million inhabitants, and only 15% exceed 20 units per million inhabitants. While the increasing availability of MRI units has revolutionized medical diagnostics, it has also introduced an unintended consequence: the accumulation of Gd in the environment, including freshwater systems.

MRI unit density, population size, and wastewater generation were also positively correlated with Gd_SN_/Gd_SN_* (Table S6), particularly for Europe and the Americas. By contrast, Oceania showed no significant relationships, likely due to limited data coverage. The global Gd mean anomaly (6.3 ± 21.3) was three times higher than the median (1.93), reflecting the influence of extreme outliers. In Europe, despite being the most homogeneous region in terms of HDI (Table S5), the Gd_SN_/Gd_SN_* anomalies showed the widest range and a skewed distribution, largely driven by exceptionally high anomalies in Germany (up to 187) and the UK (54), whereas most countries clustered around values near 2, indicating predominantly moderate to low anthropogenic signals in drinking water. The divergence between mean and median values underscores the uneven distribution of Gd contamination, with localized hotspots linked to high MRI density and potentially substantial Gd releases to the environment. In fact, while European countries, Australia, South Korea, and Canada rank among the highest globally for healthcare access, anomalies in most countries remained substantially lower than in Germany and UK. This suggests that beyond healthcare access, other factors critically shape local contamination levels.

Wastewater production emerged as a strong predictor of GdCAs contamination for most regions (Table S5), reflecting the high stability of these complexes and their poor removal in treatment systems. Advanced wastewater treatment technologies are largely confined to high-income countries with near-universal sanitation coverage, although even within these countries, treatment capacity remains uneven. At the same time, many low- and middle-income countries still lack access to basic wastewater management. Even where wastewater treatment exists, conventional wastewater plants remove less than 10% of total Gd,^14^ leading to elevated Gd anomalies in effluents.^15,32,33^ Reported effluent Gd concentrations can exceed influent levels by up to 10-fold,^32^ likely due to preferential removal of light REE, including geogenic Gd, during treatment,^34^ and possibly due to Gd release from sewage sludge,^35^ though the latter remains untested. Consequently, the relative HREE enrichment in tap waters may reflect the preferential removal of the LREE via Fe hydroxide formation during water clarification treatment^18^ or precipitation during ozonation,^36^ leading to elevated Yb/Nd and Dy/Nd ratios relative to river waters.

Advanced wastewater treatment technologies offer substantially higher removal efficiencies, with activated carbon adsorbing 70–90% of GdCAs^37^ and reverse osmosis achieving up to 99%.^38^ However, assessing treatment levels across and within regions remains challenging. Even within countries (e.g., USA, Spain, Brazil, and Australia), sanitation infrastructure and wastewater treatment technologies can differ markedly between and within cities and can fluctuate seasonally depending on operational conditions attending to different circumstances. Besides, the chemical behavior of GdCAs, including degradation pathways during wastewater treatment or in the environment, remains poorly understood. Regulatory requirements for micropollutants such as GdCAs are scarce, and heterogeneity in treatment systems likely contributes to the large country-level variability observed (Figure 4; Table S2). These factors, however, do not fully explain the observed patterns. For instance, in the United States, New York would be expected to exhibit the highest Gd_SN_/Gd_SN_* ratios given its population size, advanced healthcare infrastructure, and large wastewater production. Yet smaller cities, such as Washington, DC. and San Diego showed anomalies 7- and 2-fold higher, respectively (Figure 4; Table S2). Similarly, in Spain, Australia, and China, anticipated Gd_SN_/Gd_SN_* peaks in Madrid, Sydney, and Shanghai were substantially lower than expected, with maximum values instead occurring in smaller cities (Figure 4). These discrepancies point to the influence of local water supply sources and treatment practices on Gd distribution.

This becomes evident at finer spatial scale. Germany has both the highest MRI density,^39^ as well as the largest Gd_SN_/Gd_SN_* anomalies and Gd_AN_ concentrations. Our measurements of drinking water in Berlin in 2025 yield Gd_AN_ concentrations of 61.2 ng L^–1^, 45 higher than those in 2009 (1.36 ng L^–1^ in 2009).^18^ This apparent rise in contamination exceeds trends reported for estuaries and coastal systems receiving wastewater effluents,^28,40^ highlighting that the contamination impacts are particularly significant for drinking water supplies, while also notable in marine environments.^22,28,41^

In addition to that, and at the within-city scale, Berlin also shows substantial variability in Gd_AN_ contamination, detectable in both drinking water and tap water-based soft drinks.^18,19,21,42^ This heterogeneity cannot be explained by MRI density, wastewater production, or population size, but is instead driven by differences in water supply sources. Western districts of Berlin, relying on shallow groundwater influenced by bank filtration from contaminated rivers, show concentrations 50 times higher (Figure S2) than eastern districts, which rely on deeper, less impacted aquifers.^15,43^ Similar source-related differences may explain unexpectedly low anomalies in cities such as New York (USA, surface water from three watersheds), Sydney (Australia, surface + desalination), and Madrid (Spain, surface water with groundwater backup).

Speciation analyses showed that Dotarem (a macrocyclic compound) was the predominant species across evaluated samples, whereas Magnevist and Prohance, when detected, occurred only at concentrations below the limit of quantification (Table S4). Dotarem prevalence in samples analyzed from various cities in Europe likely reflects the decision adopted in 2017 by the European Medicines Agency (EMA) to suspend the use of linear GdCAs, except for specific applications, due to concerns over Gd retention in the brain and other tissues. This regulatory shift may also explain the change observed in German drinking water, where the previously dominant linear agent Magnevist (Gd-DTPA)^44^ has now been replaced by the macrocyclic Dotarem. The US Food & Drug Administration (FDA) similarly warns that linear GBCAs exhibit greater gadolinium retention. The predominance of macrocyclic agents in drinking water thus reflects both clinical preference and regulatory decisions. However, linear GdCAs remain in use in parts of the Americas, Japan, and Australia.

GdCAs, while considered stable compounds that provide safe conditions for patients by limiting Gd exposure and incorporation, eventually undergo degradation. Degradation was observed in samples stored for more than one year before speciation analyses, and although anomalies in REE patterns persisted, the specific agents could no longer be identified. The persistence of Gd anomalies, despite expected GdCA degradation, has previously been demonstrated in a study assessing temporal variations of Gd anomalies over a 20-year period.^28^ The pathways and rates of GdCA degradation in water remain poorly constrained and may involve bacterial activity and photo-oxidation,^45^ as well as transmetalation with other strongly binding metals.^46^ Dissociation rates are strongly influenced by complex structure.^47^ Both linear and macrocyclic GdCAs undergo acid-catalyzed dissociation; however, macrocyclic ligands exhibit dissociation half-lives several orders of magnitude longer at both neutral and acidic pH (i.e., < 5 s to 20 days at 37 °C and pH 1.2 for linear and macrocyclic GdCAs, respectively), reflecting their substantially higher kinetic stability.^48^ Despite this inertness, *in vitro* studies demonstrate high gastrointestinal bioaccessibility of macrocyclic agents, particularly Dotarem.^23^ In contrast, the less stable linear GBCAs dissociate within seconds under acidic conditions,^47^ and the released Gd­(III) tends to precipitate as oxides in the gastrointestinal tract, reducing their bioaccessibility.^23^ Under a scenario of normal to slow gastric emptying (4h of acid exposure), only 10% of the Gd_AN_ from ingested drinking water would be expected to remain.^48^ At maximum concentrations currently measured in drinking water (∼70 ng L^–1^), a daily intake of 2 L over an 85-year lifetime would correspond to a cumulative ingestion of ∼ 4.3 × 10^–3^ g of Gd. This amount represents only ∼ 0.4% of a single intravenously administered GdCA dose (1.1–1.3 g Gd).

Our findings document a clear pathway for Gd from urban sewage through rivers and groundwater into drinking water worldwide, directly linked to the use of MRI examinations and the reuse of treated wastewater, with no other anthropogenic source of Gd at this scale. Because both factors are projected to rise - namely, the increasing reliance on MRI due to its diagnostic advantages and the continued essential use of GdCAs in clinical practice, along with the growing global demand for potable water - the levels of Gd_AN_ in drinking water are expected to increase accordingly. Our results establish a critical global baseline for future assessments of human exposure and potential health impacts. While the GdCA concentrations we measured in this study do not pose immediate risks from drinking water consumption, the pathways by which these compounds could accumulate in biological tissues remain unclear.

The presence of Gd_AN_ in drinking water also illustrates what is likely occurring with other persistent, sewage-derived contaminants, such as antihypertensives, antibiotics, antidepressants,^49^ and per- and polyfluoroalkyl substances (PFAS), many of which are biologically active at low concentrations and lack established Acceptable Daily Intake (ADI) values,^50^ as is also the case for GdCAs. The multiplicity of potential interactions and synergistic effects among co-occurring contaminants, together with the difficulty of assessing how pollutant exposure intersects with dimensions of human biosocial identity, raises additional concerns for human health and underscores the urgent need for holistic evaluations of emerging contaminants in drinking water. Such evaluations should be coupled with the adoption of advanced wastewater treatment technologies, such as activated carbon, ozonation, and reverse osmosis, that achieve substantially higher removal efficiencies than conventional methods. These measures are particularly pressing in the context of population growth and climate change, which will not only exacerbate water scarcity^51,52^ but likely intensify contamination.^4,53–55^ While bank filtration, water reclamation, and reuse are essential for ensuring resilient water supply, their effectiveness will ultimately depend on the integration of advanced treatment processes with robust regulatory frameworks capable of addressing the expanding spectrum and potential health risks of emerging contaminants.

## Supplementary Material



## Data Availability

All raw data supporting this study are provided in the Supporting Information.

## Notes

Additional figures, as well as comprehensive data on individual REE concentrations and speciation results (PDF).
