# Supplementary material for: A Global Imprint of Gadolinium-Based Contrast Agents Used in Magnetic Resonance Imaging in Drinking Water
Source: Environ Sci Technol. 2026 Jun 30;60(27):19611–9. doi: 10.1021/acs.est.6c06733 (PMC13374097; doi:10.1021/acs.est.6c06733)
Supplement: Supplementary file 1 [file es6c06733_si_001.pdf]

Supplementary material

A Global Imprint of Gadolinium-Based Contrast Agents Used in Magnetic Resonance Imaging in Drinking Water

Vanessa Hatje[1,2,3*], Franciele Castro Novais[1,], Anna Maria Orani[3], Maria Elisabete Machado[1,2,], Pere Masque[4], Marc Metian[3]

[1]Centro Interdisciplinar de Energia e Ambiente (CIEnAm), Universidade Federal da Bahia, Salvador, Bahia 40170-115, Brazil.

[2]Instituto de Química, Universidade Federal da Bahia, Salvador, Bahia 40170-115, Brazil.
[3]IAEA Marine Environment Laboratories, Department of Nuclear Sciences and Applications, International Atomic Energy Agency, 98000 Monaco, Principality of Monaco
[4]School of Natural Sciences, Centre for Marine Ecosystems Research, Edith Cowan University, Joondalup,WA 6027, Australia

Corresponding author: vhatje@ufba.br & v.hatje@iaea.org

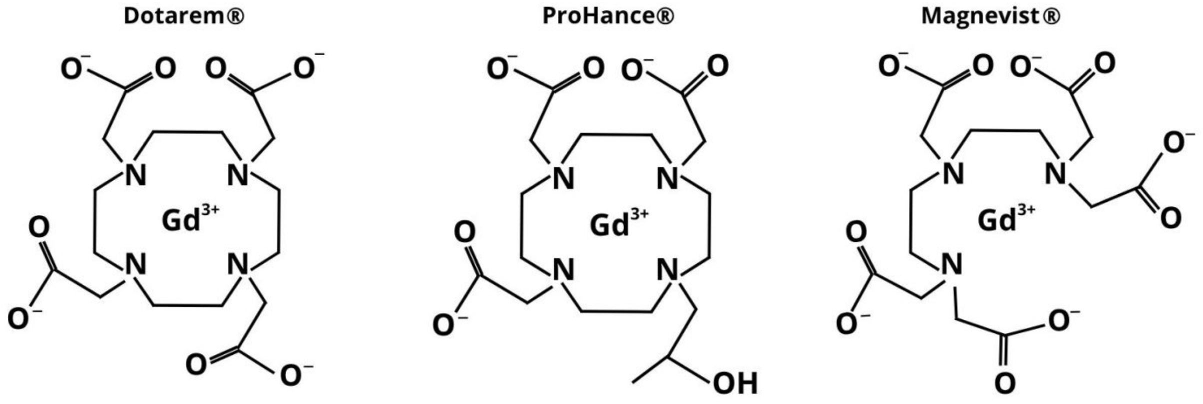

Fig. S1. Schematic representation of the molecular structures of the commercial Gd-based contrast agents: linear Magnevist (Gd-DTPA) and the macrocyclic agents Dotarem (Gd-DOTA) and ProHance (Gd-DO3A).

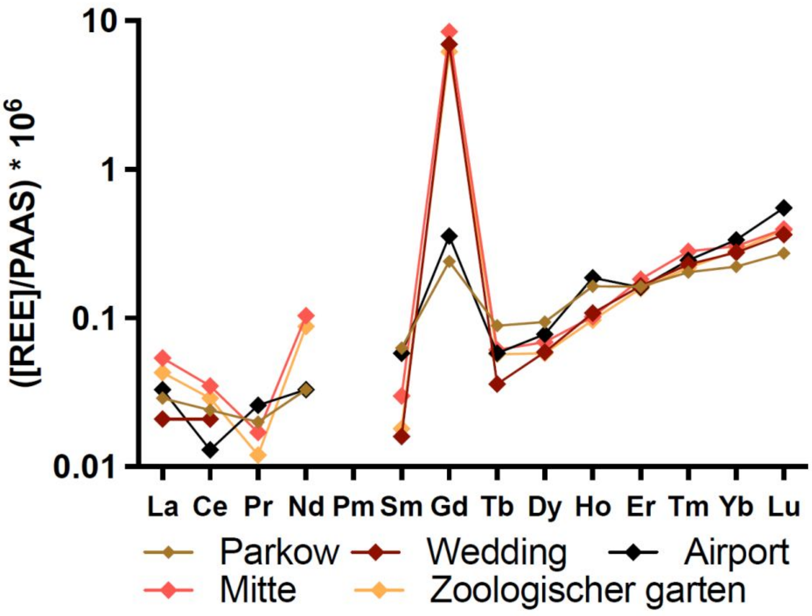

Fig. S2. PAAS-normalized REE concentrations in tap water from Berlin districts, Germany. Samples from the western districts (Mitte, Wedding, and Zoologischer Garten) show larger Gd anomalies than those from the eastern districts (Pankow, Airport). This difference reflects the contrasting drinking-water sources: in the western districts, water is produced through artificial groundwater recharge using water from the contaminated Lake Tegel and the Havel River.

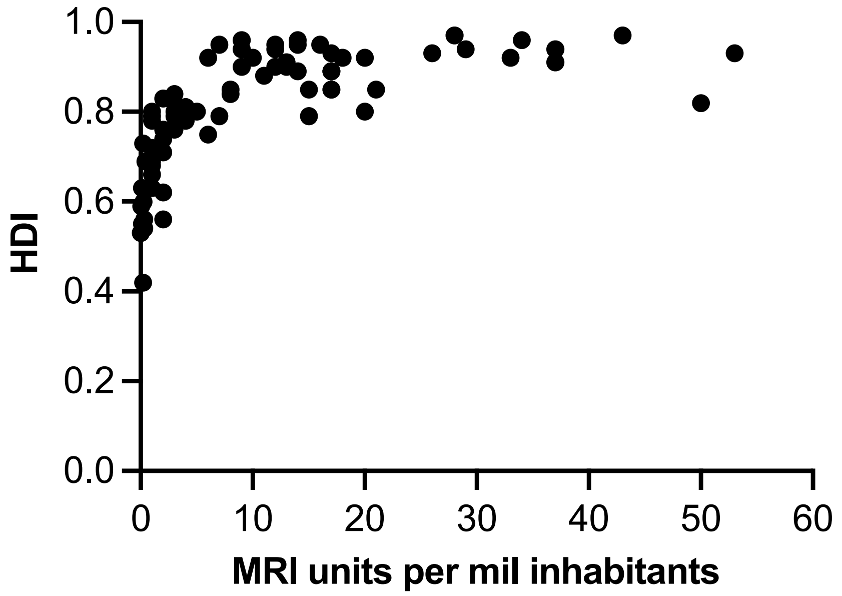

Figure S3. Availability of MRI units per 1 million inhabitants vs Human Development Index (HDI) of the country (UNITED NATIONS DEVELOPMENT PROGRAMME (UNDP). *Human Development Index*. Data Center, Human Development Reports. 2025. <https://hdr.undp.org/data-center/human-development-index).

Table S1. Main analytical features for the HPLC-ICPMS method applied for Gd speciation in freshwater samples (Orani et al. 2025-under review)

**Parameters**	**Magnevist**	**Dotarem**	**ProHance**
Working range (ng Kg^{-1} as Gd)	3-10000	3-10000	100-10000
LOD (ng Kg^{-1} as Gd)	1.1	1.1	30
LOQ (ng Kg^{-1} as Gd)	3.3	3.3	90
Repeatability	3%	3%	6 %
Intermediate precision	5%	5%	9%
Recovery (n=3)	110%	101%	105%
Expanded Uncertainty (k=2)	11%	11%	20%
Retention time (s)	332±30	542±20	1340±40

Table S2. Concentrations of REE in tap water (ng L^{-1} ± SD; n = 2), accompanied by PAAS-normalized ratios (Yb_{SN}/Nd_{SN}, Dy_{SN}/Nd_{SN}, and Dy_{SN}/Yb_{SN}), Gd anomalies (Gd_{SN}/Gd_{SN}^*), Ce anomalies ((Ce_{SN}/Ce_{SN}^*), and estimates of the geogenic (Gd_{geo}) and anthropogenic (Gd_{AN}) fractions of total Gd (ng L^{-1}).

	Germany						Croatia				Norway
**Elements**	**Berlin**	**BER01[2] (Berlin)**	**BER02[2] (Berlin)**	**BER03[2] (Berlin)**	**Bremen**	**Hamburg**	**Fazana**	**Nacice**	**Sibenick**	**Zagreb**	**Tromso**
**Y**	8.48 ± 0.17	11.5 ± 0.12	14.9 ± 0.07	18.2 ± 0.12	12.1 ± 0.01	6.28 ± 0.10	3.43 ± 0.03	2.95 ± 0.18	5.72 ± 0.18	4.71 ± 0.05	7.16 ± 0.07
**La**	4.29 ± 0.08	4.67 ± 0.10	6.52 ± 0.22	9.70 ± 0.11	3.03 ± 0.05	2.23 ± 0.05	3.09 ± 0.03	2.52 ± 0.19	3.08 ± 0.08	5.82 ± 0.04	7.99 ± 0.08
**Ce**	8.90 ± 0.65	5.95 ± 0.22	10.7 ± 0.06	17.8 ± 0.21	6.45 ± 0.55	5.09 ± 0.05	4.93 ± 0.17	6.09 ± 0.36	6.06 ± 0.15	12.43 ± 0.44	9.81 ± 0.08
**Pr**	1.05 ± 0.07	1.05 ± 0.03	2.00 ± 0.05	3.13 ± 0.01	0.54 ± 0.06	0.53 ± 0.01	0.51 ± 0.03	0.55 ± 0.05	0.46 ± 0.03	1.11 ± 0.02	0.96 ± 0.02
**Nd**	4.18 ± 0.13	4.33 ± 0.06	9.19 ± 0.09	12.4 ± 0.05	2.20 ± 0.14	3.14 ± 0.19	2.27 ± 0.08	2.34 ± 0.14	1.87 ± 0.08	4.36 ± 0.03	5.50 ± 0.06
**Sm**	0.57 ± 0.06	0.85 ± 0.05	1.89 ± 0.03	2.28 ± 0.06	0.62 ± 0.03	0.44 ± 0.01	0.41 ± 0.04	0.42 ± 0.01	0.53 ± 0.02	0.71 ± 0.03	0.20 ± 0.01
**Eu**	0.23 ± 0.03	0.16 ± 0.01	0.35 ± 0.03	0.33 ± 0.02	0.10 ± 0.01	0.20 ± 0.01	0.11 ± 0.01	0.09 ± 0.01	0.09 ± 0.01	0.16 ± 0.01	0.50 ± 0.01
**Gd**	61.5 ± 1.56	57.4 ± 0.41	12.1 ± 0.08	21.3 ± 0.14	4.92 ± 0.07	1.31 ± 0.07	0.54 ± 0.02	0.52 ± 0.02	0.48 ± 0.01	2.34 ± 0.07	1.32 ± 0.05
**Tb**	0.11 ± 0.01	0.17 ± 0.01	0.33 ± 0.02	0.36 ± 0.01	0.06 ± 0.01	0.08 ± 0.01	0.09 ± 0.01	0.08 ± 0.01	0.09 ± 0.01	0.22 ± 0.01	0.15 ± 0.01
**Dy**	0.70 ± 0.01	1.22 ± 0.07	2.13 ± 0.01	2.49 ± 0.02	0.41 ± 0.02	0.70 ± 0.04	0.82 ± 0.03	0.57 ± 0.03	0.64 ± 0.01	1.49 ± 0.01	0.76 ± 0.03
**Ho**	0.10 ± 0.01	0.31 ± 0.01	0.51 ± 0.03	0.51 ± 0.03	0.12 ± 0.01	0.17 ± 0.01	0.21 ± 0.02	0.13 ± 0.01	0.16 ± 0.01	0.37 ± 0.03	0.17 ± 0.01
**Er**	0.80 ± 0.04	1.24 ± 0.02	1.67 ± 0.02	1.94 ± 0.02	0.42 ± 0.01	0.67 ± 0.01	0.57 ± 0.06	0.43 ± 0.01	0.50 ± 0.02	1.33 ± 0.02	0.59 ± 0.01
**Tm**	0.16 ± 0.01	0.21 ± 0.01	0.25 ± 0.01	0.28 ± 0.01	0.07 ± 0.01	0.12 ± 0.01	0.10 ± 0.01	0.10 ± 0.01	0.10 ± 0.01	0.18 ± 0.01	0.10 ± 0.01
**Yb**	2.07 ± 0.05	2.47 ± 0.06	2.36 ± 0.08	2.68 ± 0.05	0.68 ± 0.03	1.18 ± 0.06	0.53 ± 0.01	0.59 ± 0.02	0.61 ± 0.02	1.19 ± 0.01	0.58 ± 0.02
**Lu**	0.32 ± 0.02	0.44 ± 0.02	0.34 ± 0.02	0.54 ± 0.01	0.13 ± 0.01	0.22 ± 0.01	0.10 ± 0.01	0.13 ± 0.01	0.15 ± 0.01	0.22 ± 0.01	0.15 ± 0.02
**∑REEY**	93.5	92.0	65.2	93.8	31.8	22.4	17.7	17.5	20.5	36.6	35.9
**∑REE**	85.0	80.5	47.6	75.6	19.8	16.1	14.3	14.6	14.8	31.9	28.8
**Yb_{SN}/Nd_{SN}**	6.14	7.07	3.18	2.69	3.83	4.65	2.92	3.14	4.05	3.38	1.30
**Dy_{SN}/Nd_{SN}**	1.18	1.97	1.63	1.41	1.32	1.56	2.53	1.72	2.39	2.40	0.97
**Dy_{SN}/Yb_{SN}**	0.19	0.28	0.51	0.52	0.34	0.34	0.87	0.55	0.59	0.71	0.74
**Ce_{SN}/Ce_{SN}^***	1.01	0.65	0.71	0.78	1.22	1.13	0.95[1]	1.24	1.22	1.18	0.85[1]
**Gd_{geo}**	0.32	0.84	1.84	2.13	0.39	0.40	0.54	0.52	0.43	1.27	0.80
**Gd_{AN}**	61.2	56.6	10.3	19.2	4.53	0.91	-	-	-	1.06	0.52
**Gd_{AN} %**	99	99	85	90	92	70	-	-	-	46	39
**Gd_{SN}/Gd_{SN}^***	187	67.9	6.57	10.0	12.6	3.24	0.97	1.00	1.00	1.81	1.63

	Spain								
**Elements**	**Barcelona**	**Calonge**	**Cartagena**	**Huelva**	**Madrid**	**Majorca**	**Puerto Real**	**Seville**	**Tona**
**Y**	26.5 ± 0.02	18.4 ± 0.44	16.4 ± 0.35	8.89 ± 0.03	16.9 ± 0.31	13.00 ± 0.05	9.99 ± 0.16	9.82 ± 0.08	33.2 ± 1.43
**La**	15.3 ± 0.05	4.87 ± 0.09	16.4 ± 0.37	6.22 ± 0.14	17.0 ± 0.21	6.68 ± 0.15	5.58 ± 0.22	6.27 ± 0.05	9.87 ± 0.26
**Ce**	24.0 ± 0.12	26.5 ± 0.42	22.4 ± 0.04	9.95 ± 0.15	21.3 ± 0.15	10.0 ± 0.53	8.79 ± 0.42	10.6 ± 0.15	20.2 ± 1.15
**Pr**	3.28 ± 0.01	0.94 ± 0.01	3.21 ± 0.04	1.76 ± 0.10	1.89 ± 0.02	1.40 ± 0.01	1.59 ± 0.05	1.96 ± 0.04	1.56 ± 0.06
**Nd**	11.8 ± 0.19	4.52 ± 0.17	13.3 ± 0.06	6.02 ± 0.21	7.10 ± 0.48	5.51 ± 0.10	4.49 ± 0.20	4.41 ± 0.16	5.24 ± 0.16
**Sm**	3.71 ± 0.09	0.76 ± 0.01	2.99 ± 0.07	1.69 ± 0.07	1.40 ± 0.06	1.47 ± 0.05	2.26 ± 0.08	1.48 ± 0.06	1.19 ± 0.05
**Eu**	0.63 ± 0.01	1.75 ± 0.04	0.53 ± 0.01	0.30 ± 0.02	0.37 ± 0.01	0.27 ± 0.01	0.40 ± 0.05	0.27 ± 0.01	1.01 ± 0.04
**Gd**	33.0 ± 0.05	2.59 ± 0.13	4.25 ± 0.09	1.45 ± 0.06	2.26 ± 0.09	1.33 ± 0.05	2.67 ± 0.14	1.71 ± 0.05	1.88 ± 0.04
**Tb**	0.46 ± 0.02	0.24 ± 0.01	0.53 ± 0.03	0.26 ± 0.01	0.25 ± 0.01	0.21 ± 0.01	0.22 ± 0.01	0.21 ± 0.01	0.23 ± 0.01
**Dy**	2.66 ± 0.09	2.39 ± 0.07	2.80 ± 0.04	1.45 ± 0.09	2.04 ± 0.04	1.24 ± 0.09	1.36 ± 0.04	1.37 ± 0.06	2.40 ± 0.14
**Ho**	0.77 ± 0.05	0.74 ± 0.01	0.68 ± 0.01	0.38 ± 0.01	0.47 ± 0.01	0.32 ± 0.02	0.43 ± 0.01	0.41 ± 0.02	1.17 ± 0.04
**Er**	2.54 ± 0.03	3.53 ± 0.06	1.69 ± 0.03	1.42 ± 0.07	1.62 ± 0.01	0.95 ± 0.02	1.18 ± 0.07	1.17 ± 0.01	4.17 ± 0.21
**Tm**	0.38 ± 0.01	0.61 ± 0.03	0.24 ± 0.01	0.23 ± 0.01	0.22 ± 0.02	0.19 ± 0.02	0.22 ± 0.01	0.19 ± 0.01	0.85 ± 0.02
**Yb**	3.74 ± 0.09	4.88 ± 0.03	1.27 ± 0.05	1.53 ± 0.05	1.35 ± 0.03	1.16 ± 0.07	1.35 ± 0.06	1.19 ± 0.05	5.81 ± 0.17
**Lu**	0.64 ± 0.01	0.81 ± 0.04	0.22 ± 0.01	0.27 ± 0.01	0.19 ± 0.01	0.19 ± 0.01	0.23 ± 0.01	0.19 ± 0.02	1.09 ± 0.09
**∑REEY**	129	73.5	86.9	41.8	74.4	44.0	40.8	41.2	89.9
**∑REE**	103	55.1	70.5	32.9	57.5	31.0	30.8	31.4	56.7
**Yb_{SN}/Nd_{SN}**	3.92	13.6	1.19	3.15	2.36	2.60	3.73	3.35	13.7
**Dy_{SN}/Nd_{SN}**	1.57	3.71	1.48	1.68	2.02	1.58	2.12	2.18	3.21
**Dy_{SN}/Yb_{SN}**	0.40	0.28	1.24	0.53	0.86	0.61	0.57	0.65	0.23
**Ce_{SN}/Ce_{SN}***	0.81	2.99	0.75	0.72	0.90[1]	0.79	0.71	0.73	1.24
**Gd_{geo}**	3.09	1.44	3.09	1.45	1.57	1.33	1.68	1.34	1.24
**Gd_{AN}**	30.0	1.15	1.16	-	0.69	-	0.99	0.37	0.66
**Gd_{AN} %**	91	56	27	-	31	-	37	22	35
**Gd_{SN}/Gd_{SN}***	10.7	2.22	1.36	1.00	1.42	1.00	1.56	1.26	1.51

	Ireland	Monaco	Austria	Portugal	Switzerland		France		
Elements	Dublin	Monaco	Vienna	Lisboa	Lausanne	Zurich	Corsica	Paris	Saint Denis
Y	13.6 ± 0.08	16.1 ± 0.21	12.8 ± 0.25	18.5 ± 0.21	13.2 ± 0.23	6.48 ± 0.28	60.1 ± 0.50	15.5 ± 0.16	5.50 ± 0.11
La	4.87 ± 0.04	1.77 ± 0.09	5.24 ± 0.10	18.6 ± 0.03	5.44 ± 0.07	1.90 ± 0.05	40.2 ± 0.42	5.41 ± 0.06	2.78 ± 0.04
Ce	8.87 ± 0.08	3.44 ± 0.08	3.38 ± 0.08	32.6 ± 0.07	9.22 ± 0.15	2.59 ± 0.19	44.2 ± 0.41	9.81 ± 0.21	5.68 ± 0.02
Pr	0.97 ± 0.05	0.31 ± 0.02	0.71 ± 0.04	3.62 ± 0.03	0.99 ± 0.07	0.30 ± 0.03	9.72 ± 0.25	1.59 ± 0.04	0.77 ± 0.02
Nd	3.57 ± 0.03	1.41 ± 0.02	2.22 ± 0.06	14.5 ± 0.09	3.99 ± 0.12	1.79 ± 0.11	36.3 ± 0.29	4.73 ± 0.12	3.50 ± 0.09
Sm	0.96 ± 0.02	0.28 ± 0.03	0.45 ± 0.02	2.54 ± 0.08	0.99 ± 0.07	0.70 ± 0.04	7.88 ± 0.12	1.19 ± 0.02	0.92 ± 0.01
Eu	0.17 ± 0.01	0.11 ± 0.01	0.11 ± 0.01	0.64 ± 0.01	0.17 ± 0.01	0.12 ± 0.01	1.25 ± 0.07	0.49 ± 0.04	0.20 ± 0.01
Gd	16.9 ± 0.09	0.35 ± 0.02	0.56 ± 0.04	3.59 ± 0.01	6.25 ± 0.08	5.04 ± 0.15	9.13 ± 0.18	8.30 ± 0.07	1.33 ± 0.04
Tb	0.16 ± 0.01	0.06 ± 0.01	0.07 ± 0.01	0.35 ± 0.01	0.14 ± 0.01	0.11 ± 0.01	1.41 ± 0.06	0.26 ± 0.02	0.12 ± 0.01
Dy	1.05 ± 0.02	0.37 ± 0.01	0.61 ± 0.03	2.15 ± 0.04	0.89 ± 0.02	0.65 ± 0.04	8.53 ± 0.11	2.02 ± 0.07	0.80 ± 0.04
Ho	0.35 ± 0.02	0.09 ± 0.01	0.13 ± 0.01	0.51 ± 0.01	0.33 ± 0.02	0.12 ± 0.01	1.86 ± 0.05	0.49 ± 0.01	0.18 ± 0.01
Er	1.14 ± 0.01	0.39 ± 0.04	0.49 ± 0.01	1.84 ± 0.01	0.96 ± 0.06	0.46 ± 0.04	5.98 ± 0.08	1.15 ± 0.05	0.64 ± 0.01
Tm	0.23 ± 0.01	0.11 ± 0.01	0.10 ± 0.01	0.25 ± 0.01	0.23 ± 0.02	0.10 ± 0.01	0.95 ± 0.05	0.19 ± 0.01	0.08 ± 0.01
Yb	1.51 ± 0.01	0.62 ± 0.01	0.68 ± 0.02	1.63 ± 0.04	1.38 ± 0.04	0.67 ± 0.05	6.12 ± 0.08	1.36 ± 0.07	0.54 ± 0.04
Lu	0.28 ± 0.01	0.11 ± 0.01	0.13 ± 0.01	0.33 ± 0.01	0.25 ± 0.02	0.11 ± 0.01	0.90 ± 0.05	0.17 ± 0.01	0.08 ± 0.01
∑REEY	54.6	25.5	27.7	102	44.4	21.2	235	52.6	23.1
∑REE	41.0	9.42	14.9	83.1	31.2	14.7	174	37.2	17.6
Yb_{SN}/Nd_{SN}	5.23	5.45	3.77	1.40	4.29	4.65	2.09	3.55	1.92
Dy_{SN}/Nd_{SN}	2.06	1.82	1.93	1.04	1.57	2.69	1.65	2.99	1.59
Dy_{SN}/Yb_{SN}	0.39	0.33	0.51	0.75	0.37	0.58	0.79	0.84	0.83
$Ce_{SN}/Ce_{SN}*$	0.98[1]	1.12	0.42	0.96[1]	0.96[1]	0.83[1]	0.54	0.81	0.93
Gd_{geo}	0.93	0.28	0.47	2.35	0.88	0.70	7.18	1.49	0.83
Gd_{AN}	15.9	-	-	1.24	5.37	4.34	1.95	6.81	0.50
Gd_{AN} %	95	-	-	35	86	86	21	82	65
$Gd_{SN}/Gd_{SN}*$	17.9	1.23	1.19	1.50	7.02	7.07	1.25	5.50	1.58

Elements	Romania	Belgium	Greece	Poland	Latvia	Lithuania	Italy		
	Bucharest	Brussels	Athens	Warsaw	Riga	Klaipeda	Milan	Rome	Vatican
**Y**	9.20 ± 0.09	7.88 ± 0.08	23.5 ± 0.12	11.8 ± 0.15	20.2 ± 0.24	14.3 ± 0.25	11.3 ± 0.04	8.79 ± 0.09	-
**La**	4.85 ± 0.03	3.61 ± 0.04	5.82 ± 0.05	5.61 ± 0.13	8.09 ± 0.19	9.22 ± 0.19	5.56 ± 0.07	5.24 ± 0.14	2.69 ± 0.07
**Ce**	8.40 ± 0.12	6.77 ± 0.12	10.3 ± 0.08	6.97 ± 0.09	8.62 ± 0.19	13.4 ± 0.23	9.03 ± 0.10	8.94 ± 0.09	8.00 ± 0.06
**Pr**	1.19 ± 0.03	1.19 ± 0.06	1.18 ± 0.01	1.56 ± 0.02	1.35 ± 0.06	1.51 ± 0.03	1.02 ± 0.07	0.81 ± 0.02	0.68 ± 0.07
**Nd**	5.70 ± 0.10	5.33 ± 0.15	4.81 ± 0.03	5.25 ± 0.05	6.04 ± 0.07	6.45 ± 0.17	4.36 ± 0.02	3.47 ± 0.09	2.36 ± 0.04
**Sm**	1.63 ± 0.02	0.98 ± 0.07	0.91 ± 0.01	0.86 ± 0.03	1.50 ± 0.06	1.00 ± 0.03	0.85 ± 0.03	1.05 ± 0.03	0.59 ± 0.08
**Eu**	0.35 ± 0.03	0.25 ± 0.02	0.20 ± 0.01	0.27 ± 0.02	0.28 ± 0.01	0.19 ± 0.01	0.24 ± 0.01	0.18 ± 0.01	0.11 ± 0.01
**Gd**	1.99 ± 0.04	2.10 ± 0.05	1.88 ± 0.05	6.83 ± 0.08	6.45 ± 0.10	2.49 ± 0.05	2.00 ± 0.04	0.93 ± 0.03	0.53 ± 0.05
**Tb**	0.18 ± 0.01	0.15 ± 0.01	0.17 ± 0.01	0.14 ± 0.01	0.25 ± 0.02	0.16 ± 0.01	0.13 ± 0.01	0.16 ± 0.01	0.07 ± 0.01
**Dy**	1.08 ± 0.02	0.94 ± 0.04	1.43 ± 0.02	1.14 ± 0.03	1.71 ± 0.06	0.95 ± 0.01	0.76 ± 0.02	0.96 ± 0.03	0.57 ± 0.04
**Ho**	0.27 ± 0.01	0.27 ± 0.01	0.45 ± 0.01	0.35 ± 0.01	0.58 ± 0.03	0.27 ± 0.01	0.24 ± 0.02	0.24 ± 0.01	0.13 ± 0.01
**Er**	0.73 ± 0.03	0.79 ± 0.03	1.57 ± 0.02	1.26 ± 0.06	1.71 ± 0.10	0.89 ± 0.01	0.69 ± 0.04	1.02 ± 0.02	0.57 ± 0.04
**Tm**	0.14 ± 0.01	0.13 ± 0.01	0.36 ± 0.01	0.19 ± 0.01	0.30 ± 0.02	0.21 ± 0.01	0.12 ± 0.01	0.15 ± 0.01	0.07 ± 0.01
**Yb**	0.83 ± 0.06	0.75 ± 0.01	2.58 ± 0.02	1.82 ± 0.03	1.74 ± 0.09	1.30 ± 0.02	0.87 ± 0.03	1.00 ± 0.02	0.56 ± 0.03
**Lu**	0.15 ± 0.01	0.12 ± 0.01	0.66 ± 0.01	0.28 ± 0.01	0.27 ± 0.03	0.24 ± 0.01	0.12 ± 0.01	0.16 ± 0.01	0.08 ± 0.01
**∑REEY**	36.7	31.3	55.8	44.3	59.1	52.5	37.2	33.1	-
**∑REE**	27.5	23.4	32.3	32.5	38.9	38.3	26.0	24.3	17.1
**Yb_{SN}/Nd_{SN}**	1.80	1.75	6.64	4.29	3.58	2.38	2.48	3.56	2.92
**Dy_{SN}/Nd_{SN}**	1.33	1.23	2.08	1.52	1.98	0.98	1.23	1.94	1.68
**Dy_{SN}/Yb_{SN}**	0.74	0.71	0.31	0.35	0.55	0.41	0.50	0.54	0.58
**Ce_{SN}/Ce_{SN}***	0.84[1]	0.79	0.94[1]	0.57	0.63	0.90[1]	0.92[1]	1.05	1.43
**Gd_{geo}**	1.43	0.92	0.94	0.89	1.68	0.87	0.75	0.97	0.53
**Gd_{AN}**	0.56	1.18	0.94	5.99	4.77	1.62	1.25	-	-
**Gd_{AN} %**	28	56	50	88	74	65	63	-	-
**Gd_{SN}/Gd_{SN}***	1.37	2.25	1.97	8.06	3.78	2.82	2.64	0.95	1.00

Elements	United Kingdom		Denmark	Slovenia	Albania	Czech Republic	Estonia	Ukraine	Cyprus	Georgia
	London (1)	London (2)	Copenhagen	Ljubljana	Tirana	Rimov	Tallinn	Kiev	Nicosia	Tbilisi
**Y**	9.99 ± 0.02	7.02 ± 0.01	40.2 ± 0.08	5.27 ± 0.09	8.92 ± 0.12	18.1 ± 0.09	5.75 ± 0.01	11.3 ± 0.25	6.56 ± 0.15	17.5 ± 0.14
**La**	1.17 ± 0.03	3.76 ± 0.09	30.2 ± 0.09	0.68 ± 0.01	2.93 ± 0.09	4.90 ± 0.06	11.8 ± 0.06	7.27 ± 0.16	4.65 ± 0.11	6.14 ± 0.14
**Ce**	2.27 ± 0.02	3.30 ± 0.02	43.6 ± 0.11	0.96 ± 0.01	2.73 ± 0.04	7.12 ± 0.10	14.1 ± 0.06	9.74 ± 0.19	8.47 ± 0.12	9.57 ± 0.13
**Pr**	0.20 ± 0.01	0.31 ± 0.02	4.99 ± 0.09	0.34 ± 0.02	0.59 ± 0.01	1.02 ± 0.07	2.05 ± 0.06	0.90 ± 0.04	1.04 ± 0.05	1.30 ± 0.01
**Nd**	1.08 ± 0.01	1.56 ± 0.01	16.9 ± 0.04	1.35 ± 0.04	2.70 ± 0.08	4.22 ± 0.05	7.16 ± 0.03	3.39 ± 0.03	4.53 ± 0.09	5.30 ± 0.05
**Sm**	0.17 ± 0.01	0.19 ± 0.02	2.86 ± 0.04	0.32 ± 0.01	1.35 ± 0.09	1.53 ± 0.04	1.22 ± 0.07	0.71 ± 0.03	1.41 ± 0.06	0.98 ± 0.02
**Eu**	0.15 ± 0.02	0.14 ± 0.02	0.51 ± 0.03	0.07 ± 0.01	0.27 ± 0.02	0.29 ± 0.02	0.24 ± 0.01	0.13 ± 0.01	0.25 ± 0.02	0.23 ± 0.01
**Gd**	30.5 ± 0.10	19.7 ± 0.18	3.36 ± 0.08	0.53 ± 0.03	1.65 ± 0.08	2.67 ± 0.04	2.73 ± 0.01	1.21 ± 0.08	1.40 ± 0.01	1.45 ± 0.08
**Tb**	0.17 ± 0.01	0.12 ± 0.01	0.38 ± 0.02	0.10 ± 0.01	0.21 ± 0.01	0.35 ± 0.01	0.13 ± 0.01	0.12 ± 0.01	0.18 ± 0.01	0.16 ± 0.01
**Dy**	0.88 ± 0.05	0.72 ± 0.04	2.32 ± 0.08	0.63 ± 0.01	1.29 ± 0.07	2.13 ± 0.03	0.77 ± 0.01	0.80 ± 0.04	1.13 ± 0.04	1.25 ± 0.05
**Ho**	0.16 ± 0.01	0.17 ± 0.02	0.68 ± 0.04	0.15 ± 0.01	0.26 ± 0.01	0.47 ± 0.01	0.18 ± 0.01	0.30 ± 0.01	0.25 ± 0.01	0.37 ± 0.03
**Er**	1.03 ± 0.05	0.93 ± 0.04	2.08 ± 0.09	0.48 ± 0.01	0.79 ± 0.03	1.79 ± 0.01	0.60 ± 0.04	0.94 ± 0.01	0.84 ± 0.03	1.06 ± 0.09
**Tm**	0.23 ± 0.01	0.17 ± 0.02	0.32 ± 0.02	0.09 ± 0.01	0.14 ± 0.01	0.28 ± 0.01	0.14 ± 0.01	0.15 ± 0.01	0.19 ± 0.01	0.24 ± 0.01
**Yb**	1.77 ± 0.05	2.34 ± 0.02	2.25 ± 0.08	0.62 ± 0.02	0.98 ± 0.05	1.79 ± 0.01	0.91 ± 0.01	1.11 ± 0.04	1.28 ± 0.06	1.57 ± 0.06
**Lu**	0.36 ± 0.01	0.47 ± 0.02	0.32 ± 0.02	0.10 ± 0.01	0.19 ± 0.01	0.30 ± 0.02	0.14 ± 0.01	0.14 ± 0.01	0.20 ± 0.01	0.25 ± 0.01
**∑REEY**	50.1	40.5	151	11.7	25.0	47.0	47.9	38.2	32.4	47.4
**∑REE**	40.1	33.5	111	6.45	16.1	28.9	42.1	26.9	25.8	29.9
**Yb_{SN}/Nd_{SN}**	20.4	18.6	1.64	5.72	4.50	5.27	1.57	4.05	3.50	3.66
**Dy_{SN}/Nd_{SN}**	5.75	3.24	0.96	3.27	3.35	3.54	0.76	1.64	1.75	1.65
**Dy_{SN}/Yb_{SN}**	0.28	0.17	0.58	0.57	0.74	0.67	0.48	0.41	0.50	0.34
**$Ce_{SN}/Ce_{SN}*$**	1.14	0.74	0.85	0.48	0.50	0.77[1]	0.69	0.92	0.93[1]	0.82
**Gd_{geo}**	0.56	0.40	2.59	0.53	1.46	2.12	1.31	0.79	1.20	0.94
**Gd_{AN}**	29.9	0.10	0.77	-	-	0.56	1.43	0.42	0.20	0.51
**Gd_{AN} %**	98	17	23	-	-	21	52	35	15	35
**$Gd_{SN}/Gd_{SN}*$**	53.5	48.3	1.28	1.00	1.12	1.25	2.06	1.51	1.15	1.52

Elements	Brazil						
	BSB[2] (Brasilia)	RJ[2] (Rio de Janeiro)	Rio de Janeiro (1)	Rio de Janeiro (2)	Rio de Janeiro (3)	SSA[2] (Salvador)	São Paulo
**Y**	6.65 ± 0.04	76.3 ± 0.46	66.7 ± 0.21	50.4 ± 0.52	73.1 ± 0.24	6.64 ± 0.48	18.1 ± 0.23
**La**	9.68 ± 0.06	101 ± 0.47	89.8 ± 0.20	77.2 ± 0.69	98.6 ± 0.21	9.29 ± 0.23	14.9 ± 0.15
**Ce**	11.9 ± 0.07	194 ± 0.56	117 ± 0.54	102 ± 0.61	170 ± 1.08	12.6 ± 0.13	25.1 ± 0.12
**Pr**	1.39 ± 0.04	33.5 ± 0.65	18.8 ± 0.10	14.4 ± 0.02	18.7 ± 0.53	2.27 ± 0.16	2.58 ± 0.02
**Nd**	6.76 ± 0.04	115 ± 0.48	73.6 ± 0.23	64.4 ± 0.72	77.3 ± 0.01	9.14 ± 0.09	12.5 ± 0.18
**Sm**	1.38 ± 0.05	13.3 ± 0.32	9.02 ± 0.13	7.83 ± 0.02	10.5 ± 0.61	1.99 ± 0.11	2.25 ± 0.10
**Eu**	0.19 ± 0.01	7.44 ± 0.25	1.65 ± 0.02	1.56 ± 0.08	1.84 ± 0.02	0.47 ± 0.01	0.39 ± 0.02
**Gd**	3.20 ± 0.23	58.9 ± 0.22	17.5 ± 0.01	9.12 ± 0.16	9.78 ± 0.01	2.71 ± 0.13	3.39 ± 0.06
**Tb**	0.19 ± 0.01	2.31 ± 0.09	0.98 ± 0.01	1.19 ± 0.01	1.37 ± 0.03	0.29 ± 0.02	0.31 ± 0.02
**Dy**	1.00 ± 0.04	20.3 ± 0.29	9.46 ± 0.01	6.17 ± 0.33	8.83 ± 0.12	1.79 ± 0.09	2.25 ± 0.02
**Ho**	0.22 ± 0.01	4.53 ± 0.15	2.04 ± 0.10	1.42 ± 0.04	1.85 ± 0.02	0.38 ± 0.02	0.46 ± 0.02
**Er**	0.64 ± 0.01	15.5 ± 0.41	7.03 ± 0.14	4.64 ± 0.07	6.52 ± 0.14	1.12 ± 0.06	1.37 ± 0.09
**Tm**	0.10 ± 0.01	5.22 ± 0.11	1.12 ± 0.01	0.56 ± 0.02	0.93 ± 0.01	0.19 ± 0.01	0.24 ± 0.01
**Yb**	0.75 ± 0.03	17.9 ± 0.16	6.19 ± 0.09	4.27 ± 0.17	6.35 ± 0.12	1.13 ± 0.03	1.65 ± 0.03
**Lu**	0.15 ± 0.01	5.68 ± 0.15	1.16 ± 0.02	0.72 ± 0.01	1.06 ± 0.01	0.21 ± 0.02	0.27 ± 0.01
**∑REEY**	44.6	672	422	347	487	50.2	85.8
**∑REE**	37.9	596	356	296	414	43.6	67.7
**Yb_{SN}/Nd_{SN}**	1.36	1.90	1.04	0.82	1.02	1.51	1.63
**Dy_{SN}/Nd_{SN}**	1.06	1.23	0.90	0.67	0.80	1.37	1.26
**Dy_{SN}/Yb_{SN}**	0.65	0.65	0.86	0.82	0.79	0.91	0.77
**Ce_{SN}/Ce_{SN}***	0.78[1]	0.81	0.69	0.74	0.96	0.66[1]	0.98[1]
**Gd_{geo}**	1.26	13.7	8.15	7.45	9.39	1.93	2.08
**Gd_{AN}**	1.94	45.2	9.32	-	-	0.78	1.31
**Gd_{AN} %**	61	77	53	-	-	29	38
**Gd_{SN}/Gd_{SN}***	2.53	4.31	2.11	1.21	1.03	1.41	1.60

	Colombia	Peru	Chile	Ecuador	
**Elements**	**Santa Marta**	**Lima**	**Santiago**	**Guayaquil**	**Quito**
**Y**	39.9 ± 0.24	8.05 ± 0.09	12.3 ± 0.21	21.1 ± 0.05	13.4 ± 0.18
**La**	16.9 ± 0.15	5.74 ± 0.09	3.98 ± 0.08	14.9 ± 0.10	9.42 ± 0.15
**Ce**	12.2 ± 0.11	7.60 ± 0.09	7.54 ± 0.10	17.6 ± 0.09	12.5 ± 0.06
**Pr**	2.42 ± 0.10	1.00 ± 0.05	0.64 ± 0.01	3.20 ± 0.02	2.29 ± 0.10
**Nd**	10.7 ± 0.16	4.48 ± 0.10	2.49 ± 0.05	17.3 ± 0.10	9.16 ± 0.10
**Sm**	2.37 ± 0.05	0.19 ± 0.02	0.45 ± 0.02	3.94 ± 0.05	2.95 ± 0.10
**Eu**	0.48 ± 0.02	0.75 ± 0.03	0.09 ± 0.02	0.95 ± 0.08	0.71 ± 0.06
**Gd**	4.62 ± 0.02	1.17 ± 0.05	0.53 ± 0.02	4.23 ± 0.03	2.95 ± 0.08
**Tb**	0.33 ± 0.01	0.17 ± 0.01	0.07 ± 0.01	0.65 ± 0.03	0.46 ± 0.03
**Dy**	2.16 ± 0.06	0.82 ± 0.04	0.48 ± 0.01	3.59 ± 0.02	2.53 ± 0.12
**Ho**	0.52 ± 0.03	0.19 ± 0.01	0.13 ± 0.01	0.85 ± 0.02	0.60 ± 0.01
**Er**	2.15 ± 0.09	0.61 ± 0.01	0.57 ± 0.02	2.51 ± 0.08	1.76 ± 0.03
**Tm**	0.45 ± 0.01	0.12 ± 0.01	0.08 ± 0.01	0.39 ± 0.01	0.27 ± 0.0
**Yb**	2.40 ± 0.01	0.81 ± 0.03	0.89 ± 0.03	2.10 ± 0.04	1.61 ± 0.09
**Lu**	0.50 ± 0.01	0.20 ± 0.01	0.18 ± 0.04	0.37 ± 0.03	0.28 ± 0.02
**∑REEY**	98.1	31.9	30.4	93.6	61.0
**∑REE**	58.2	23.9	18.1	72.5	47.5
**Yb_{SN}/Nd_{SN}**	2.77	2.25	4.43	1.50	2.18
**Dy_{SN}/Nd_{SN}**	1.41	1.29	1.36	1.46	1.93
**Dy_{SN}/Yb_{SN}**	0.51	0.57	0.31	0.97	0.89
**$Ce_{SN}/Ce_{SN}*$**	0.46	0.76[1]	1.13	0.61[1]	0.65
**Gd_{geo}**	2.40	0.86	0.45	4.04	2.95
**Gd_{AN}**	2.22	0.31	-	-	-
**Gd_{AN} %**	48	27	-	-	-
**$Gd_{SN}/Gd_{SN}*$**	1.90	1.35	1.16	1.03	1.00

Elements	United States of America							
	Atlanta	Charleston	Charlotte	Columbia	Greenville	Milwaukee	New York (1)	New York (2)
**Y**	17.7 ± 0.19	12.7 ± 0.11	11.4 ± 0.10	5.67 ± 0.06	10.7 ± 0.20	2.97 ± 0.05	10.7 ± 0.12	49.4 ± 1.00
**La**	9.30 ± 0.06	8.92 ± 0.09	10.3 ± 0.18	6.08 ± 0.07	10.6 ± 0.22	2.41 ± 0.12	13.5 ± 0.14	36.4 ± 0.90
**Ce**	13.6 ± 0.21	14.7 ± 0.15	17.4 ± 0.21	7.50 ± 0.07	9.06 ± 0.15	3.93 ± 0.10	21.3 ± 0.15	45.7 ± 0.87
**Pr**	1.11 ± 0.03	2.36 ± 0.10	2.56 ± 0.01	1.38 ± 0.01	2.50 ± 0.06	0.46 ± 0.03	2.71 ± 0.08	9.40 ± 0.18
**Nd**	7.37 ± 0.10	8.80 ± 0.09	9.63 ± 0.10	4.91 ± 0.08	9.08 ± 0.02	1.99 ± 0.06	11.2 ± 0.15	37.7 ± 0.91
**Sm**	2.07 ± 0.10	2.16 ± 0.06	2.51 ± 0.07	1.18 ± 0.06	1.90 ± 0.01	0.77 ± 0.04	1.91 ± 0.09	10.5 ± 0.40
**Eu**	0.62 ± 0.01	0.36 ± 0.03	0.32 ± 0.01	0.21 ± 0.01	0.32 ± 0.02	0.16 ± 0.01	0.45 ± 0.03	2.44 ± 0.16
**Gd**	3.69 ± 0.07	3.16 ± 0.15	3.92 ± 0.09	3.68 ± 0.04	1.61 ± 0.05	0.72 ± 0.03	4.41 ± 0.08	11.5 ± 0.55
**Tb**	0.26 ± 0.01	0.28 ± 0.02	0.33 ± 0.01	0.13 ± 0.01	0.23 ± 0.02	0.11 ± 0.01	0.30 ± 0.01	1.61 ± 0.09
**Dy**	2.11 ± 0.02	1.77 ± 0.07	2.15 ± 0.03	0.93 ± 0.08	1.36 ± 0.04	0.64 ± 0.01	1.74 ± 0.10	8.86 ± 0.09
**Ho**	0.55 ± 0.01	0.36 ± 0.01	0.45 ± 0.01	0.31 ± 0.02	0.29 ± 0.03	0.14 ± 0.01	0.47 ± 0.01	2.10 ± 0.05
**Er**	2.32 ± 0.07	1.39 ± 0.09	2.04 ± 0.01	1.50 ± 0.05	1.04 ± 0.06	0.42 ± 0.01	2.25 ± 0.02	5.27 ± 0.04
**Tm**	0.46 ± 0.02	0.23 ± 0.01	0.33 ± 0.01	0.21 ± 0.01	0.20 ± 0.01	0.08 ± 0.01	0.49 ± 0.01	0.85 ± 0.05
**Yb**	2.53 ± 0.03	1.58 ± 0.04	2.56 ± 0.01	1.58 ± 0.02	1.13 ± 0.01	0.54 ± 0.02	5.76 ± 0.08	5.64 ± 0.10
**Lu**	0.49 ± 0.02	0.25 ± 0.01	0.37 ± 0.01	0.28 ± 0.02	0.17 ± 0.01	0.10 ± 0.01	1.51 ± 0.04	1.15 ± 0.07
**∑REEY**	64.1	58.9	66.3	35.6	50.2	15.4	78.6	229
**∑REE**	46.5	46.3	54.9	29.9	39.5	12.5	68.0	179
**Yb_{SN}/Nd_{SN}**	4.26	2.22	3.29	3.99	1.54	3.39	1.86	1.86
**Dy_{SN}/Nd_{SN}**	2.01	1.41	1.57	1.32	1.05	2.24	1.65	1.65
**Dy_{SN}/Yb_{SN}**	0.47	0.63	0.48	0.33	0.68	0.66	0.89	0.89
**Ce_{SN}/Ce_{SN}***	1.02	0.77	0.81	0.62	0.42	0.90[1]	0.85[1]	0.60[1]
**Gd_{geo}**	2.73	0.70	2.12	0.94	1.61	0.69	1.41	10.4
**Gd_{AN}**	0.97	2.46	1.80	2.73	-	-	3.00	-
**Gd_{AN} %**	26	78	46	74	-	-	68	-
**Gd_{SN}/Gd_{SN}***	1.36	4.45	1.82	3.84	1.00	1.02	3.08	1.11

Elements	United States of America						Canada	
	Sacramento	San Diego	San Francisco	Santa Cruz	Washington DC	Woods Hole	Montreal	Ottawa
Y	10.7 ± 0.15	11.5 ± 0.17	69.7 ± 0.61	3.15 ± 0.03	10.5 ± 0.12	2.47 ± 0.07	13.0 ± 0.21	51.9 ± 0.12
La	13.4 ± 0.16	5.77 ± 0.14	112 ± 0.95	2.06 ± 0.06	3.93 ± 0.02	2.13 ± 0.04	17.4 ± 0.09	42.2 ± 0.12
Ce	21.8 ± 0.13	6.93 ± 0.03	205 ± 0.97	4.87 ± 0.11	4.08 ± 0.10	2.48 ± 0.05	16.7 ± 0.21	59.8 ± 0.16
Pr	2.81 ± 0.09	0.44 ± 0.01	21.9 ± 0.21	0.55 ± 0.01	0.74 ± 0.06	0.40 ± 0.01	1.51 ± 0.07	9.39 ± 0.10
Nd	10.4 ± 0.16	1.93 ± 0.04	80.4 ± 0.12	2.18 ± 0.05	4.53 ± 0.07	1.29 ± 0.09	5.87 ± 0.24	34.6 ± 0.14
Sm	1.65 ± 0.04	1.09 ± 0.06	12.7 ± 0.13	0.46 ± 0.04	0.97 ± 0.05	0.27 ± 0.01	0.99 ± 0.05	6.72 ± 0.08
Eu	0.38 ± 0.01	0.27 ± 0.01	2.71 ± 0.01	0.10 ± 0.01	0.32 ± 0.01	0.08 ± 0.01	0.27 ± 0.01	2.13 ± 0.04
Gd	4.50 ± 0.12	4.62 ± 0.15	12.6 ± 0.06	0.96 ± 0.03	29.3 ± 0.04	0.51 ± 0.01	2.30 ± 0.03	7.64 ± 0.07
Tb	0.31 ± 0.01	0.15 ± 0.01	1.67 ± 0.03	0.07 ± 0.01	0.29 ± 0.01	0.07 ± 0.01	0.14 ± 0.01	1.09 ± 0.02
Dy	2.18 ± 0.10	0.92 ± 0.04	9.07 ± 0.05	0.41 ± 0.01	1.94 ± 0.02	0.50 ± 0.01	0.98 ± 0.03	5.64 ± 0.09
Ho	0.58 ± 0.02	0.22 ± 0.01	2.34 ± 0.03	0.09 ± 0.01	0.64 ± 0.02	0.11 ± 0.01	0.31 ± 0.01	1.46 ± 0.02
Er	2.10 ± 0.05	1.05 ± 0.07	7.17 ± 0.03	0.26 ± 0.02	3.00 ± 0.06	0.53 ± 0.03	0.95 ± 0.04	4.29 ± 0.05
Tm	0.84 ± 0.05	0.22 ± 0.01	1.29 ± 0.01	0.04 ± 0.01	0.54 ± 0.01	0.14 ± 0.01	0.22 ± 0.01	0.71 ± 0.01
Yb	6.33 ± 0.04	1.38 ± 0.05	6.97 ± 0.10	0.28 ± 0.01	5.13 ± 0.05	1.18 ± 0.05	1.54 ± 0.02	3.96 ± 0.05
Lu	1.65 ± 0.02	0.25 ± 0.01	1.26 ± 0.01	0.05 ± 0.01	0.98 ± 0.02	0.27 ± 0.01	0.27 ± 0.02	0.59 ± 0.02
∑REEY	79.7	36.7	547	15.5	66.9	12.5	62.5	232
∑REE	68.9	25.2	478	12.4	56.3	9.97	49.5	180
Yb_{SN}/Nd_{SN}	7.57	8.88	1.07	1.57	14.0	11.4	3.26	1.42
Dy_{SN}/Nd_{SN}	1.48	3.32	0.79	1.31	3.01	2.69	1.17	1.14
Dy_{SN}/Yb_{SN}	0.20	0.37	0.74	0.84	0.21	0.24	0.36	0.80
Ce_{SN}/Ce_{SN}*	0.86	1.05	0.99	1.10	0.58[1]	0.64	0.79[1]	0.72
Gd_{geo}	1.16	0.85	10.8	0.44	1.41	0.17	0.86	6.56
Gd_{AN}	3.34	3.77	-	0.52	27.8	0.34	1.45	-
Gd_{AN} %	74	82	-	54	95	66	62	-
Gd_{SN}/Gd_{SN}*		5.36	1.15	2.14	20.5	2.90	2.65	1.15

	Jamaica	Trinidad and Tobago	Bahamas	Nicaragua	Sint Maarten	Mexico		
**Elements**	**Kingston**	**Chaguaramas**	**Nassau**	**Managua**	**Simpson Bay**	**Hermosillo**	**Mazatlán**	**Mexico City**
**Y**	14.5 ± 0.18	30.9 ± 0.21	7.27 ± 0.04	8.64 ± 0.04	12.5 ± 0.26	9.72 ± 0.09	51.0 ± 0.24	9.72 ± 0.14
**La**	10.3 ± 0.06	28.9 ± 0.12	9.99 ± 0.07	15.3 ± 0.10	10.2 ± 0.26	16.3 ± 0.15	65.2 ± 0.31	16.3 ± 0.15
**Ce**	17.3 ± 0.01	66.4 ± 0.25	12.4 ± 0.14	21.7 ± 0.15	16.2 ± 0.28	21.6 ± 0.21	117 ± 0.51	21.6 ± 0.16
**Pr**	2.42 ± 0.05	8.16 ± 0.12	1.43 ± 0.04	1.73 ± 0.03	2.29 ± 0.16	2.99 ± 0.08	17.9 ± 0.12	2.99 ± 0.08
**Nd**	9.79 ± 0.07	41.2 ± 0.22	4.86 ± 0.10	7.00 ± 0.02	8.32 ± 0.12	8.09 ± 0.01	64.2 ± 0.21	8.09 ± 0.01
**Sm**	3.53 ± 0.08	8.54 ± 0.15	0.75 ± 0.01	0.98 ± 0.03	1.46 ± 0.06	1.31 ± 0.04	8.57 ± 0.10	1.31 ± 0.04
**Eu**	0.79 ± 0.02	1.96 ± 0.08	0.16 ± 0.01	0.18 ± 0.01	0.56 ± 0.02	0.23 ± 0.01	1.62 ± 0.03	0.23 ± 0.01
**Gd**	5.36 ± 0.09	8.65 ± 0.05	0.80 ± 0.01	1.53 ± 0.02	1.42 ± 0.01	1.75 ± 0.07	11.7 ± 0.10	1.75 ± 0.07
**Tb**	0.84 ± 0.01	1.24 ± 0.03	0.11 ± 0.01	0.12 ± 0.01	0.19 ± 0.01	0.18 ± 0.01	1.77 ± 0.04	0.18 ± 0.01
**Dy**	5.03 ± 0.02	6.51 ± 0.10	0.68 ± 0.03	0.79 ± 0.01	1.00 ± 0.03	1.10 ± 0.06	8.57 ± 0.17	1.10 ± 0.06
**Ho**	1.34 ± 0.09	1.05 ± 0.01	0.21 ± 0.01	0.23 ± 0.01	0.24 ± 0.02	0.29 ± 0.01	1.72 ± 0.09	0.29 ± 0.01
**Er**	4.27 ± 0.12	3.38 ± 0.08	0.60 ± 0.03	0.76 ± 0.01	0.65 ± 0.02	1.07 ± 0.05	5.13 ± 0.08	1.07 ± 0.05
**Tm**	0.93 ± 0.01	0.38 ± 0.02	0.12 ± 0.01	0.15 ± 0.01	0.09 ± 0.01	0.18 ± 0.01	0.78 ± 0.01	0.18 ± 0.01
**Yb**	4.95 ± 0.07	2.76 ± 0.10	0.83 ± 0.01	1.04 ± 0.01	0.47 ± 0.01	1.29 ± 0.09	4.34 ± 0.07	1.29 ± 0.09
**Lu**	1.26 ± 0.01	0.40 ± 0.01	0.13 ± 0.01	0.14 ± 0.01	0.11 ± 0.01	0.24 ± 0.02	0.68 ± 0.04	0.24 ± 0.02
**∑REEY**	82.6	210	40.4	60.3	55.6	66.3	360	66.3
**∑REE**	68.2	179	33.1	51.6	43.2	56.6	309	56.6
**Yb_{SN}/Nd_{SN}**	6.26	0.83	2.09	1.84	0.70	2.11	0.84	1.97
**Dy_{SN}/Nd_{SN}**	3.60	1.11	0.96	0.79	0.84	1.67	0.94	0.95
**Dy_{SN}/Yb_{SN}**	0.58	1.33	0.46	0.43	1.20	0.79	1.12	0.48
**$Ce_{SN}/Ce_{SN}*$**	0.83	1.04	0.79	1.02	0.81	0.76	0.83	0.75
**Gd_{geo}**	4.35	8.10	0.80	0.82	1.38	1.11	11.1	0.91
**Gd_{AN}**	-	-	-	0.71	-	-	-	0.84
**Gd_{AN} %**	-	-	-	47	-	-	-	48
**$Gd_{SN}/Gd_{SN}*$**	1.22	1.05	1.00	1.85	1.02	0.97	1.04	1.90

	Panama	Costa Rica	Antiqua e Barbuda	Saint Vincent and the Grenadines	Guatemala	El Salvador	Cuba	
Elements	Miraflores	San Jose	Antiqua e Barbuda	Kingstown	Guatemala City	San Salvador	Habana	Cienfuegos
Y	19.3 ± 0.29	14.1 ± 0.12	3.30 ± 0.03	19.8 ± 0.23	4.78 ± 0.26	50.4 ± 0.67	8.47 ± 0.12	12.0 ± 0.11
La	12.2 ± 0.23	8.35 ± 0.01	9.20 ± 0.10	6.92 ± 0.15	2.50 ± 0.06	10.7 ± 0.10	6.36 ± 0.06	13.4 ± 0.18
Ce	17.4 ± 0.25	10.4 ± 0.15	14.7 ± 0.23	7.98 ± 0.18	5.77 ± 0.01	12.9 ± 0.09	9.58 ± 0.06	17.8 ± 0.17
Pr	3.40 ± 0.01	2.07 ± 0.01	1.81 ± 0.18	2.02 ± 0.05	0.82 ± 0.02	2.89 ± 0.06	0.89 ± 0.07	2.06 ± 0.09
Nd	12.6 ± 0.29	9.31 ± 0.01	6.60 ± 0.05	8.98 ± 0.03	3.11 ± 0.02	10.8 ± 0.16	3.15 ± 0.13	8.23 ± 0.07
Sm	2.24 ± 0.12	2.30 ± 0.09	1.35 ± 0.04	1.77 ± 0.03	0.78 ± 0.03	2.74 ± 0.12	0.62 ± 0.04	0.98 ± 0.06
Eu	0.50 ± 0.02	0.43 ± 0.01	0.22 ± 0.01	0.50 ± 0.01	0.16 ± 0.01	0.50 ± 0.01	0.15 ± 0.01	0.16 ± 0.01
Gd	3.32 ± 0.05	2.25 ± 0.05	1.14 ± 0.01	2.12 ± 0.04	1.96 ± 0.01	4.74 ± 0.08	1.12 ± 0.08	1.33 ± 0.06
Tb	0.40 ± 0.01	0.33 ± 0.02	0.21 ± 0.01	0.34 ± 0.02	0.11 ± 0.01	0.65 ± 0.01	0.13 ± 0.0	0.14 ± 0.01
Dy	3.26 ± 0.09	1.93 ± 0.02	1.22 ± 0.03	2.14 ± 0.14	0.67 ± 0.01	4.20 ± 0.11	0.73 ± 0.01	1.23 ± 0.04
Ho	0.68 ± 0.02	0.40 ± 0.02	0.26 ± 0.01	0.54 ± 0.05	0.16 ± 0.01	1.03 ± 0.04	0.18 ± 0.01	0.34 ± 0.01
Er	2.34 ± 0.01	1.16 ± 0.02	0.82 ± 0.03	1.60 ± 0.06	0.58 ± 0.04	4.70 ± 0.01	0.55 ± 0.01	0.94 ± 0.05
Tm	0.41 ± 0.03	0.18 ± 0.02	0.13 ± 0.01	0.26 ± 0.03	0.10 ± 0.01	0.72 ± 0.01	0.13 ± 0.01	0.15 ± 0.01
Yb	2.06 ± 0.08	1.14 ± 0.05	0.93 ± 0.05	1.73 ± 0.01	0.78 ± 0.02	4.73 ± 0.03	0.94 ± 0.01	0.98 ± 0.02
Lu	0.32 ± 0.02	0.17 ± 0.01	0.14 ± 0.01	0.26 ± 0.01	0.11 ± 0.01	0.83 ± 0.01	0.16 ± 0.02	0.14 ± 0.01
∑REEY	80.5	54.5	42.05	56.9	22.4	113	33.1	59.8
∑REE	61.2	40.4	38.8	37.2	17.6	62.1	24.7	47.9
Yb_{SN}/Nd_{SN}	2.03	1.52	1.74	2.38	3.12	5.41	3.69	1.47
Dy_{SN}/Nd_{SN}	1.82	1.46	1.29	1.67	1.50	2.72	1.62	1.05
Dy_{SN}/Yb_{SN}	0.90	0.96	0.74	0.70	0.48	0.50	0.44	0.71
$Ce_{SN}/Ce_{SN}*$	0.65	0.61	0.87	0.51	0.97	0.56	0.97[1]	0.81[1]
Gd_{geo}	2.56	2.03	1.21	1.95	0.97	3.67	0.69	0.98
Gd_{AN}	0.76	-	-	-	0.98	1.07	0.43	0.34
Gd_{AN} %	23	-	-	-	50	23	38	26
$Gd_{SN}/Gd_{SN}*$	1.28	1.09	0.95	1.07	1.99	1.27	1.59	1.33

	Zimbabwe	Senegal	Algeria	Mali	Togo		South Africa	
**Elements**	**Harare**	**Dakar**	**Algiers**	**Bamako**	**Kara**	**Lome**	**Johannesburg**	**Port Elizabeth**
**Y**	4.35 ± 0.03	14.8 ± 0.23	10.5 ± 0.25	9.59 ± 0.10	21.3 ± 0.31	85.8 ± 0.66	9.35 ± 0.10	77.7 ± 0.39
**La**	23.7 ± 0.15	23.0 ± 0.28	5.18 ± 0.03	12.0 ± 0.12	44.3 ± 0.40	60.2 ± 0.54	9.26 ± 0.04	101 ± 2.11
**Ce**	64.3 ± 0.31	37.0 ± 0.21	10.5 ± 0.21	23.3 ± 0.21	75.1 ± 0.18	306 ± 1.02	11.5 ± 0.37	147 ± 1.89
**Pr**	4.09 ± 0.09	4.94 ± 0.11	1.12 ± 0.08	3.16 ± 0.08	7.78 ± 0.21	47.4 ± 0.28	1.80 ± 0.02	28.3 ± 0.47
**Nd**	15.4 ± 0.12	16.9 ± 0.25	4.35 ± 0.13	12.5 ± 0.15	26.8 ± 0.21	323 ± 1.09	5.50 ± 0.06	102 ± 1.06
**Sm**	1.80 ± 0.01	3.96 ± 0.15	1.15 ± 0.03	1.54 ± 0.01	4.04 ± 0.18	122 ± 0.98	0.86 ± 0.10	20.2 ± 0.57
**Eu**	0.37 ± 0.01	0.66 ± 0.02	0.17 ± 0.01	0.31 ± 0.01	0.77 ± 0.01	29.2 ± 0.11	0.17 ± 0.01	3.68 ± 0.01
**Gd**	2.96 ± 0.06	3.12 ± 0.06	1.42 ± 0.07	2.79 ± 0.07	5.22 ± 0.03	136 ± 0.87	5.54 ± 0.08	21.5 ± 0.04
**Tb**	0.27 ± 0.01	0.48 ± 0.03	0.18 ± 0.01	0.27 ± 0.01	0.46 ± 0.04	19.2 ± 0.12	0.16 ± 0.01	2.88 ± 0.04
**Dy**	1.58 ± 0.01	2.31 ± 0.04	1.13 ± 0.06	1.64 ± 0.07	2.71 ± 0.07	103 ± 0.89	1.25 ± 0.02	17.3 ± 0.11
**Ho**	0.33 ± 0.01	0.51 ± 0.03	0.24 ± 0.01	0.32 ± 0.01	0.64 ± 0.02	20.6 ± 0.14	0.25 ± 0.01	3.80 ± 0.01
**Er**	1.02 ± 0.06	1.47 ± 0.04	0.73 ± 0.03	0.94 ± 0.02	1.93 ± 0.01	55.6 ± 0.24	1.06 ± 0.01	11.1 ± 0.03
**Tm**	0.23 ± 0.01	0.22 ± 0.01	0.13 ± 0.01	0.15 ± 0.01	0.29 ± 0.01	6.92 ± 0.10	0.21 ± 0.01	1.73 ± 0.09
**Yb**	1.62 ± 0.07	1.55 ± 0.03	0.84 ± 0.03	0.97 ± 0.05	2.11 ± 0.06	49.5 ± 0.29	1.37 ± 0.02	11.4 ± 0.43
**Lu**	0.32 ± 0.01	0.27 ± 0.01	0.12 ± 0.01	0.14 ± 0.01	0.36 ± 0.02	8.60 ± 0.22	0.19 ± 0.01	1.68 ± 0.10
**∑REEY**	122	111	37.7	69.6	194	1374	48.5	552
**∑REE**	118	96.4	27.2	60.1	173	1288	39.1	474
**Yb_{SN}/Nd_{SN}**	1.31	1.14	3.39	0.96	0.97	1.90	3.10	1.38
**Dy_{SN}/Nd_{SN}**	0.72	0.96	1.83	0.91	0.71	2.24	1.60	1.19
**Dy_{SN}/Yb_{SN}**	0.55	0.84	0.76	0.95	0.73	1.18	0.52	0.86
**Ce_{SN}/Ce_{SN}***	1.57	0.83	1.05	0.91	0.97[1]	1.38	0.68	0.66
**Gd_{geo}**	1.55	3.04	1.11	1.41	3.18	122	0.79	18.6
**Gd_{AN}**	1.41	-	0.30	0.85	2.04	-	4.75	-
**Gd_{AN} %**	48	-	21	30	39	-	86	-
**Gd_{SN}/Gd_{SN}***	1.88	1.01	1.25	1.41	1.62	1.10	6.94	1.14

Elements	Chad<br>Ndjamena	Nigeria<br>Benin	Seychelles<br>Mahe Island	Tunisia<br>Tunis	Kenya<br>Mombasa	Reunion Island<br>Reunion Island	Ghana<br>Accra	Zambia<br>Lusaka
**Y**	15.6 ± 0.04	442 ± 9.09	561 ± 2.24	29.1 ± 0.59	14.5 ± 0.05	7.01 ± 0.08	37.6 ± 0.55	537 ± 2.08
**La**	1.06 ± 0.01	360 ± 3.01	374 ± 6.45	14.6 ± 0.12	7.91 ± 0.01	3.91 ± 0.03	34.3 ± 0.54	378 ± 2.85
**Ce**	2.76 ± 0.09	944 ± 10.0	845 ± 2.46	22.1 ± 0.23	9.75 ± 0.11	5.79 ± 0.06	23.7 ± 0.15	881 ± 9.25
**Pr**	0.24 ± 0.01	184 ± 1.20	119 ± 3.56	3.01 ± 0.15	1.68 ± 0.05	1.23 ± 0.03	7.06 ± 0.19	102 ± 2.98
**Nd**	1.59 ± 0.08	933 ± 4.38	460 ± 4.44	13.0 ± 0.27	7.68 ± 0.10	3.14 ± 0.03	27.8 ± 0.26	399 ± 4.01
**Sm**	0.61 ± 0.01	209 ± 1.05	97.2 ± 0.10	3.13 ± 0.06	1.69 ± 0.08	0.68 ± 0.03	4.66 ± 0.07	86.1 ± 1.09
**Eu**	0.10 ± 0.01	43.7 ± 0.25	18.4 ± 0.09	0.79 ± 0.01	0.28 ± 0.01	0.11 ± 0.01	1.21 ± 0.09	19.8 ± 0.86
**Gd**	0.45 ± 0.01	204 ± 2.47	111 ± 2.54	8.40 ± 0.02	2.90 ± 0.16	0.75 ± 0.01	5.14 ± 0.06	122 ± 4.80
**Tb**	0.09 ± 0.01	27.3 ± 0.79	17.2 ± 0.52	0.41 ± 0.03	0.21 ± 0.01	0.11 ± 0.01	0.78 ± 0.02	20.3 ± 0.79
**Dy**	0.48 ± 0.01	164 ± 2.01	107 ± 2.01	2.44 ± 0.06	1.79 ± 0.07	0.69 ± 0.04	4.75 ± 0.03	138 ± 2.95
**Ho**	0.11 ± 0.01	33.4 ± 0.09	23.0 ± 0.25	0.53 ± 0.06	0.38 ± 0.01	0.14 ± 0.01	0.98 ± 0.01	34.1 ± 0.20
**Er**	0.35 ± 0.01	97.9 ± 0.36	67.9 ± 0.67	1.61 ± 0.06	2.04 ± 0.01	0.33 ± 0.02	2.94 ± 0.14	101 ± 4.52
**Tm**	0.06 ± 0.01	14.9 ± 0.17	10.7 ± 0.21	0.27 ± 0.01	0.39 ± 0.01	0.06 ± 0.01	0.44 ± 0.01	16.1 ± 0.16
**Yb**	0.42 ± 0.01	99.3 ± 0.69	71.6 ± 0.94	1.21 ± 0.01	3.95 ± 0.04	0.39 ± 0.01	2.93 ± 0.01	113 ± 2.30
**Lu**	0.08 ± 0.01	14.3 ± 0.30	11.7 ± 0.39	0.18 ± 0.02	1.13 ± 0.02	0.05 ± 0.01	0.44 ± 0.01	16.3 ± 0.19
**∑REEY**	24.0	3770	2894	101	56.3	24.4	155	2964
**∑REE**	8.39	3329	2333	71.7	41.8	17.4	117	2427
**Yb_{SN}/Nd_{SN}**	3.28	1.32	1.93	1.15	6.37	1.56	1.31	3.50
**Dy_{SN}/Nd_{SN}**	2.12	1.23	1.63	1.32	1.63	1.54	1.20	2.42
**Dy_{SN}/Yb_{SN}**	0.65	0.93	0.84	1.14	0.26	0.99	0.92	0.69
**$Ce_{SN}/Ce_{SN}*$**	1.33	0.88	0.97	0.80[1]	0.64	0.64	0.37	1.08
**Gd_{geo}**	0.45	188	103	2.80	1.51	0.59	4.77	116
**Gd_{AN}**	-	-	-	5.60	1.40	0.16	-	-
**Gd_{AN} %**	-	-	-	67	48	21	-	-
**$Gd_{SN}/Gd_{SN}*$**	1.00	1.07	1.05	2.96	1.90	1.25	1.06	1.04

	India	Singapore	Turkey	Saudi Arabia	Qatar	Indonesia	
**Elements**	**Ahmedabad**	**Singapore**	**Izmir**	**Thuwal**	**Doha**	**Jakarta**	**Tangerang**
**Y**	93.3 ± 0.30	14.8 ± 0.10	71.9 ± 0.67	14.1 ± 0.05	121 ± 0.11	183 ± 0.34	1144 ± 38.01
**La**	37.6 ± 0.39	15.3 ± 0.12	28.7 ± 0.53	9.27 ± 0.05	167 ± 2.48	59.5 ± 0.88	649 ± 10.39
**Ce**	63.4 ± 0.10	21.6 ± 0.08	33.4 ± 0.03	13.3 ± 0.17	353 ± 4.01	119 ± 2.01	945 ± 5.31
**Pr**	7.78 ± 0.43	3.46 ± 0.08	5.81 ± 0.07	1.89 ± 0.01	38.9 ± 0.09	25.7 ± 0.19	148 ± 1.95
**Nd**	32.5 ± 0.57	13.4 ± 0.08	29.2 ± 0.39	6.63 ± 0.13	143 ± 1.21	107 ± 0.72	623 ± 5.23
**Sm**	7.14 ± 0.21	2.41 ± 0.03	6.88 ± 0.09	1.28 ± 0.05	31.6 ± 0.44	30.9 ± 0.30	126 ± 3.56
**Eu**	1.53 ± 0.09	0.52 ± 0.01	1.35 ± 0.09	0.22 ± 0.01	5.51 ± 0.09	7.18 ± 0.18	27.9 ± 0.89
**Gd**	8.38 ± 0.11	3.17 ± 0.03	11.3 ± 0.21	1.14 ± 0.06	27.7 ± 0.03	33.1 ± 0.29	144 ± 1.01
**Tb**	1.49 ± 0.07	0.44 ± 0.03	1.79 ± 0.12	0.16 ± 0.01	4.04 ± 0.01	5.29 ± 0.07	21.6 ± 0.75
**Dy**	9.70 ± 0.03	2.35 ± 0.07	10.7 ± 0.49	1.19 ± 0.02	22.6 ± 0.53	33.3 ± 0.31	141 ± 0.38
**Ho**	2.31 ± 0.07	0.41 ± 0.02	2.36 ± 0.08	0.30 ± 0.01	4.76 ± 0.25	7.20 ± 0.01	40.2 ± 0.55
**Er**	6.93 ± 0.04	1.71 ± 0.09	6.96 ± 0.05	0.77 ± 0.03	12.3 ± 0.37	20.7 ± 0.35	149 ± 2.02
**Tm**	1.04 ± 0.07	0.34 ± 0.02	1.13 ± 0.03	0.17 ± 0.01	1.81 ± 0.06	2.75 ± 0.04	26.3 ± 0.19
**Yb**	5.45 ± 0.14	2.24 ± 0.14	7.76 ± 0.16	0.98 ± 0.01	9.59 ± 0.33	19.3 ± 0.31	198 ± 0.26
**Lu**	0.92 ± 0.07	0.36 ± 0.01	1.28 ± 0.02	0.11 ± 0.01	1.53 ± 0.05	3.10 ± 0.05	40.9 ± 0.15
**∑REEY**	279	82.5	221	51.5	945	657	4425
**∑REE**	186	67.7	149	37.4	824	474	3281
**Yb_{SN}/Nd_{SN}**	2.08	2.07	3.29	1.82	0.83	2.23	3.94
**Dy_{SN}/Nd_{SN}**	2.09	1.23	2.56	1.26	1.10	2.18	1.58
**Dy_{SN}/Yb_{SN}**	1.01	0.59	0.78	0.69	1.33	0.98	0.40
**Ce_{SN}/Ce_{SN}^***	0.89[1]	0.71	0.62[1]	0.77	1.06	0.73	0.73
**Gd_{geo}**	8.38	2.44	9.45	1.14	27.2	33.1	127
**Gd_{AN}**	-	0.74	-	-	-	-	-
**Gd_{AN} %**	-	23	-	-	-	-	-
**Gd_{SN}/Gd_{SN}^***	0.95	1.29	1.18	1.00	1.01	1.00	1.12

Elements	Armenia	Kuwait	Thailand	Israel	Nepal		
	Lerevan	Kuwait	Bangkok	Beer Sheva	Kathmandu	Lalitpur (1)	Lalitpur (2)
**Y**	64.6 ± 0.37	160 ± 0.87	34.9 ± 0.26	9.54 ± 0.07	3.40 ± 0.07	5.88 ± 0.14	3.28 ± 0.14
**La**	9.68 ± 0.38	81.2 ± 0.35	18.9 ± 0.11	8.50 ± 0.20	4.40 ± 0.18	4.56 ± 0.04	2.08 ± 0.04
**Ce**	12.7 ± 0.27	135 ± 0.51	34.7 ± 0.18	13.4 ± 0.25	5.85 ± 0.11	3.58 ± 0.05	5.55 ± 0.40
**Pr**	1.57 ± 0.03	18.4 ± 0.12	2.93 ± 0.03	1.50 ± 0.07	0.98 ± 0.09	0.43 ± 0.02	0.46 ± 0.02
**Nd**	7.41 ± 0.18	78.4 ± 0.24	8.64 ± 0.04	5.16 ± 0.09	3.06 ± 0.03	2.12 ± 0.06	2.35 ± 0.01
**Sm**	3.97 ± 0.03	16.2 ± 0.11	0.93 ± 0.03	0.86 ± 0.50	0.45 ± 0.03	0.22 ± 0.01	0.35 ± 0.01
**Eu**	0.76 ± 0.01	3.53 ± 0.01	0.16 ± 0.01	0.17 ± 0.01	0.13 ± 0.01	0.10 ± 0.01	0.14 ± 0.01
**Gd**	5.24 ± 0.07	18.4 ± 0.15	1.58 ± 0.03	1.50 ± 0.03	0.60 ± 0.01	0.47 ± 0.01	0.66 ± 0.01
**Tb**	0.58 ± 0.02	2.73 ± 0.06	0.11 ± 0.01	0.11 ± 0.01	0.11 ± 0.01	0.06 ± 0.01	0.09 ± 0.01
**Dy**	4.34 ± 0.10	17.3 ± 0.22	0.73 ± 0.02	0.83 ± 0.03	0.69 ± 0.04	0.31 ± 0.01	0.54 ± 0.01
**Ho**	1.10 ± 0.01	4.38 ± 0.06	0.30 ± 0.01	0.25 ± 0.03	0.17 ± 0.01	0.08 ± 0.01	0.13 ± 0.01
**Er**	4.97 ± 0.10	14.6 ± 0.20	1.54 ± 0.03	0.87 ± 0.02	0.57 ± 0.03	0.29 ± 0.02	0.53 ± 0.10
**Tm**	1.16 ± 0.04	2.26 ± 0.04	0.29 ± 0.01	0.17 ± 0.01	0.08 ± 0.01	0.04 ± 0.01	0.08 ± 0.01
**Yb**	8.03 ± 0.35	15.2 ± 0.06	1.86 ± 0.03	1.02 ± 0.08	0.69 ± 0.06	0.30 ± 0.02	0.74 ± 0.09
**Lu**	2.52 ± 0.17	2.78 ± 0.04	0.28 ± 0.01	0.15 ± 0.01	0.15 ± 0.01	0.06 ± 0.01	0.20 ± 0.01
**∑REEY**	129	570	108	44.1	21.3	18.5	17.2
**∑REE**	64.1	410	72.9	34.5	17.9	12.6	13.9
**Yb_{SN}/Nd_{SN}**	13.4	2.67	2.44	1.57	2.80	1.75	3.93
**Dy_{SN}/Nd_{SN}**	4.11	0.59	1.13	1.24	1.57	1.01	1.62
**Dy_{SN}/Yb_{SN}**	0.31	0.22	0.46	0.80	0.56	0.57	0.41
**$Ce_{SN}/Ce_{SN}*$**	0.79[1]	1.12	0.91	0.87[1]	0.68	0.61[1]	1.37
**Gd_{geo}**	3.88	0.64	0.63	3.16	0.65	0.32	0.64
**Gd_{AN}**	1.35	0.94	0.87	-	-	0.15	-
**Gd_{AN} %**	26	60	58	-	-	32	-
**$Gd_{SN}/Gd_{SN}*$**	1.33	2.48	2.36	1.20	0.91	1.44	1.04

Elements	Japan				China			
	Hiroshima	Okinawa	Tokyo	Yokosuka	Beijing	Qingdao	Shanghai	Zhoushan
Y	9.95 ± 0.59	3.75 ± 0.03	8.10 ± 0.04	5.45 ± 0.13	-	16.4 ± 0.15	9.13 ± 0.03	13.5 ± 0.15
La	2.69 ± 0.07	4.23 ± 0.01	1.01 ± 0.03	2.16 ± 0.12	2.37 ± 0.01	16.7 ± 0.21	5.99 ± 0.16	7.70 ± 0.05
Ce	5.87 ± 0.23	5.58 ± 0.12	3.12 ± 0.06	3.67 ± 0.10	8.30 ± 0.37	12.7 ± 0.31	8.36 ± 0.04	11.6 ± 0.12
Pr	0.54 ± 0.02	0.95 ± 0.04	0.26 ± 0.02	0.70 ± 0.01	0.70 ± 0.03	2.41 ± 0.13	1.82 ± 0.11	2.22 ± 0.06
Nd	2.27 ± 0.03	3.85 ± 0.21	0.73 ± 0.05	2.98 ± 0.08	1.90 ± 0.02	8.67 ± 0.22	3.81 ± 0.09	5.07 ± 0.05
Sm	0.39 ± 0.02	0.89 ± 0.01	0.61 ± 0.02	0.42 ± 0.01	0.39 ± 0.01	1.67 ± 0.01	0.99 ± 0.03	1.61 ± 0.05
Eu	0.10 ± 0.01	0.13 ± 0.01	0.11 ± 0.01	0.15 ± 0.01	0.11 ± 0.01	0.29 ± 0.01	0.27 ± 0.02	0.34 ± 0.01
Gd	0.75 ± 0.01	0.77 ± 0.04	1.81 ± 0.10	0.79 ± 0.02	0.55 ± 0.01	7.63 ± 0.32	2.20 ± 0.12	3.82 ± 0.04
Tb	0.15 ± 0.01	0.10 ± 0.01	0.08 ± 0.01	0.07 ± 0.01	0.05 ± 0.01	0.24 ± 0.01	0.14 ± 0.01	0.22 ± 0.01
Dy	1.46 ± 0.03	0.79 ± 0.03	0.57 ± 0.02	0.44 ± 0.02	0.39 ± 0.03	1.62 ± 0.09	0.86 ± 0.01	1.44 ± 0.02
Ho	0.48 ± 0.02	0.26 ± 0.01	0.22 ± 0.01	0.16 ± 0.01	0.08 ± 0.01	0.37 ± 0.01	0.29 ± 0.01	0.51 ± 0.01
Er	2.89 ± 0.06	1.40 ± 0.06	1.20 ± 0.03	0.76 ± 0.02	0.28 ± 0.02	1.14 ± 0.04	1.05 ± 0.04	2.26 ± 0.03
Tm	0.56 ± 0.01	0.26 ± 0.01	0.30 ± 0.02	0.16 ± 0.01	0.06 ± 0.01	0.26 ± 0.01	0.20 ± 0.01	0.38 ± 0.01
Yb	4.45 ± 0.05	2.30 ± 0.05	3.01 ± 0.07	2.39 ± 0.11	0.55 ± 0.02	1.96 ± 0.17	1.27 ± 0.03	3.17 ± 0.02
Lu	0.93 ± 0.05	0.44 ± 0.02	0.79 ± 0.03	0.58 ± 0.03	0.08 ± 0.01	0.41 ± 0.01	0.16 ± 0.01	0.53 ± 0.01
∑REEY	33.5	25.7	21.9	20.9	-	72.5	36.5	54.4
∑REE	23.5	21.9	13.8	15.4	16.6	56.1	27.4	40.9
Yb_{SN}/Nd_{SN}	24.3	7.40	50.8	9.91	3.61	2.81	4.13	7.73
Dy_{SN}/Nd_{SN}	4.50	1.44	5.43	1.04	1.44	1.31	1.58	1.99
Dy_{SN}/Yb_{SN}	0.19	0.20	0.11	0.10	0.40	0.47	0.38	0.26
$Ce_{SN}/Ce_{SN}*$	1.17	0.67	1.47	0.72	1.56	0.48	0.61	0.68
Gd_{geo}	0.45	0.59	0.24	0.35	0.29	1.44	0.81	1.07
Gd_{AN}	0.30	0.18	1.57	0.44	0.25	6.19	1.40	2.75
Gd_{AN} %	40	23	87	55	47	81	63	67
$Gd_{SN}/Gd_{SN}*$	1.64	1.28	7.58	2.22	1.84	5.22	2.70	3.01

Elements	Iran	Malaysia				
	Tehran	Ipoh	Johor Bahru	Kota Kinabalu	Kuala Lumpur	Sepang
**Y**	5.44 ± 0.23	185 ± 0.83	3.13 ± 0.18	10.9 ± 0.15	17.1 ± 0.12	21.3 ± 0.12
**La**	4.60 ± 0.14	125 ± 2.27	5.07 ± 0.20	5.67 ± 0.07	6.98 ± 0.09	26.3 ± 0.33
**Ce**	8.13 ± 0.25	160 ± 2.13	8.47 ± 0.12	9.04 ± 0.54	11.4 ± 0.37	45.9 ± 0.34
**Pr**	0.87 ± 0.03	29.9 ± 1.02	1.01 ± 0.03	0.96 ± 0.03	1.75 ± 0.05	5.84 ± 0.10
**Nd**	4.73 ± 0.12	113 ± 0.30	4.55 ± 0.07	5.85 ± 0.16	6.93 ± 0.12	21.1 ± 0.15
**Sm**	1.01 ± 0.01	20.0 ± 0.82	0.85 ± 0.06	1.11 ± 0.01	1.26 ± 0.09	3.96 ± 0.03
**Eu**	0.22 ± 0.01	3.61 ± 0.16	0.14 ± 0.01	0.19 ± 0.01	0.22 ± 0.01	1.57 ± 0.08
**Gd**	1.29 ± 0.01	21.8 ± 0.08	0.82 ± 0.01	1.32 ± 0.05	1.48 ± 0.01	4.69 ± 0.04
**Tb**	0.18 ± 0.01	3.05 ± 0.05	0.10 ± 0.01	0.15 ± 0.01	0.16 ± 0.01	0.52 ± 0.01
**Dy**	1.08 ± 0.04	18.7 ± 0.44	0.67 ± 0.01	1.07 ± 0.02	1.15 ± 0.01	2.72 ± 0.02
**Ho**	0.28 ± 0.01	3.87 ± 0.01	0.15 ± 0.01	0.21 ± 0.01	0.27 ± 0.01	0.71 ± 0.01
**Er**	0.91 ± 0.03	11.8 ± 0.11	0.74 ± 0.02	0.78 ± 0.03	1.20 ± 0.03	2.08 ± 0.01
**Tm**	0.16 ± 0.01	1.79 ± 0.11	0.12 ± 0.01	0.13 ± 0.01	0.22 ± 0.01	0.32 ± 0.01
**Yb**	1.06 ± 0.03	12.3 ± 0.68	0.85 ± 0.01	0.80 ± 0.01	1.61 ± 0.01	2.22 ± 0.04
**Lu**	0.18 ± 0.01	1.80 ± 0.06	0.13 ± 0.01	0.13 ± 0.01	0.23 ± 0.02	0.32 ± 0.01
**∑REEY**	30.1	711	26.8	38.3	51.9	140
**∑REE**	24.7	527	23.7	27.4	34.8	118
**Yb_{SN}/Nd_{SN}**	2.78	1.36	2.30	1.70	2.87	2.41
**Dy_{SN}/Nd_{SN}**	1.59	1.16	1.03	1.28	1.17	1.54
**Dy_{SN}/Yb_{SN}**	0.57	0.86	0.45	0.75	0.41	0.64
**$Ce_{SN}/Ce_{SN}*$**	0.98[1]	0.63	0.90[1]	0.93[1]	0.78	0.84
**Gd_{geo}**	1.10	19.7	0.70	1.21	1.16	16.9
**Gd_{AN}**	-	-	-	-	0.32	-
**Gd_{AN} %**	-	-	-	-	21	-
**$Gd_{SN}/Gd_{SN}*$**	1.16	1.09	1.15	1.08	1.26	1.08

Elements	Pakistan	Bangladesh	Syria	Bahrain	Korea	
	Islamabad	Dhaka	Damascus	Manama	Busan	Seoul
**Y**	21.3 ± 0.12	6.64 ± 0.09	16.2 ± 0.27	75.4 ± 0.42	6.57 ± 0.28	4.47 ± 0.20
**La**	26.3 ± 0.33	5.28 ± 0.13	13.7 ± 0.08	35.1 ± 0.05	1.62 ± 0.07	1.05 ± 0.03
**Ce**	45.9 ± 0.34	5.75 ± 0.04	26.5 ± 0.49	26.4 ± 0.30	3.18 ± 0.09	2.41 ± 0.02
**Pr**	5.84 ± 0.10	0.84 ± 0.01	3.82 ± 0.02	7.03 ± 0.09	0.29 ± 0.01	0.26 ± 0.01
**Nd**	21.1 ± 0.15	3.49 ± 0.05	14.7 ± 0.01	25.2 ± 0.24	1.58 ± 0.12	0.96 ± 0.03
**Sm**	3.96 ± 0.03	0.81 ± 0.01	3.67 ± 0.03	7.12 ± 0.10	0.86 ± 0.01	0.37 ± 0.01
**Eu**	1.57 ± 0.08	0.14 ± 0.01	0.60 ± 0.01	1.33 ± 0.03	0.16 ± 0.01	0.11 ± 0.01
**Gd**	4.69 ± 0.04	0.89 ± 0.03	3.80 ± 0.01	7.11 ± 0.15	4.01 ± 0.01	7.07 ± 0.01
**Tb**	0.52 ± 0.01	0.11 ± 0.01	0.48 ± 0.01	1.08 ± 0.01	0.12 ± 0.01	0.10 ± 0.01
**Dy**	2.72 ± 0.02	0.74 ± 0.03	2.90 ± 0.03	7.22 ± 0.26	0.87 ± 0.03	0.72 ± 0.01
**Ho**	0.71 ± 0.01	0.17 ± 0.01	0.60 ± 0.01	1.48 ± 0.02	0.32 ± 0.01	0.24 ± 0.02
**Er**	2.08 ± 0.01	0.48 ± 0.03	1.69 ± 0.03	4.53 ± 0.05	1.57 ± 0.01	1.17 ± 0.03
**Tm**	0.32 ± 0.01	0.09 ± 0.01	0.27 ± 0.02	0.59 ± 0.01	0.34 ± 0.01	0.33 ± 0.03
**Yb**	2.22 ± 0.04	0.63 ± 0.01	1.76 ± 0.01	2.85 ± 0.05	2.27 ± 0.03	2.15 ± 0.01
**Lu**	0.32 ± 0.01	0.10 ± 0.01	0.28 ± 0.01	0.43 ± 0.01	0.41 ± 0.02	0.54 ± 0.03
**∑REEY**	140	26.1	91.0	203	24.2	22.0
**∑REE**	118	19.5	74.7	128	17.6	17.5
**Yb_{SN}/Nd_{SN}**	1.30	2.23	1.48	1.40	17.8	27.7
**Dy_{SN}/Nd_{SN}**	0.91	1.48	1.38	2.00	3.87	5.28
**Dy_{SN}/Yb_{SN}**	0.70	0.67	0.93	1.43	0.22	0.19
**Ce_{SN}/Ce_{SN}***	0.89	0.66	0.88	0.41	1.11	1.10
**Gd_{geo}**	3.27	0.67	3.27	7.25	1.55	2.41
**Gd_{AN}**	1.42	0.21	-	-	2.46	4.66
**Gd_{AN} %**	30	24	-	-	61	66
**Gd_{SN}/Gd_{SN}***	1.42	1.30	1.15	0.97	2.55	2.89

	Australia							French Polynesia	New Zealand
**Elements**	**Adelaide**	**Brisbane**	**Canberra**	**Melbourne**	**Perth**	**Sydney**	**Tasmania**	**French Polynesia**	**Auckland**
**Y**	6.69 ± 0.17	50.9 ± 0.15	33.6 ± 0.14	100 ± 2.92	13.7 ± 0.11	-	22.4 ± 0.18	3.32 ± 0.02	34.7 ± 0.77
**La**	7.99 ± 0.01	22.3 ± 0.24	21.3 ± 0.19	69.5 ± 0.71	13.6 ± 0.11	2.62 ± 0.01	14.1 ± 0.07	4.42 ± 0.12	31.5 ± 0.15
**Ce**	9.52 ± 0.08	27.4 ± 0.32	36.3 ± 0.11	86.4 ± 0.25	6.45 ± 0.08	8.29 ± 0.70	14.8 ± 0.31	11.1 ± 0.55	40.8 ± 0.22
**Pr**	1.27 ± 0.04	4.36 ± 0.02	4.53 ± 0.03	17.9 ± 0.12	1.45 ± 0.09	0.76 ± 0.01	2.72 ± 0.03	1.42 ± 0.12	7.85 ± 0.02
**Nd**	5.44 ± 0.21	16.6 ± 0.01	17.9 ± 0.10	71.6 ± 0.41	5.50 ± 0.32	2.76 ± 0.03	10.4 ± 0.28	2.82 ± 0.01	28.7 ± 0.04
**Sm**	1.27 ± 0.04	3.55 ± 0.11	4.04 ± 0.04	15.3 ± 0.29	1.01 ± 0.07	0.70 ± 0.07	2.13 ± 0.16	0.40 ± 0.03	6.29 ± 0.02
**Eu**	0.22 ± 0.01	0.82 ± 0.01	0.75 ± 0.03	4.30 ± 0.25	0.18 ± 0.01	0.14 ± 0.01	0.39 ± 0.01	0.06 ± 0.01	1.22 ± 0.02
**Gd**	1.67 ± 0.03	4.42 ± 0.06	3.96 ± 0.01	16.3 ± 0.07	4.19 ± 0.22	0.85 ± 0.01	2.27 ± 0.02	0.41 ± 0.02	5.47 ± 0.03
**Tb**	0.17 ± 0.01	0.71 ± 0.01	0.63 ± 0.02	2.59 ± 0.01	0.15 ± 0.01	0.10 ± 0.01	0.39 ± 0.01	0.06 ± 0.01	0.87 ± 0.03
**Dy**	1.05 ± 0.03	4.21 ± 0.15	3.75 ± 0.02	15.4 ± 0.26	1.02 ± 0.07	0.82 ± 0.03	2.76 ± 0.04	0.32 ± 0.02	5.61 ± 0.29
**Ho**	0.24 ± 0.01	0.95 ± 0.02	0.81 ± 0.01	3.34 ± 0.11	0.20 ± 0.02	0.13 ± 0.01	0.61 ± 0.02	0.08 ± 0.01	1.13 ± 0.01
**Er**	0.74 ± 0.01	2.93 ± 0.05	2.78 ± 0.24	9.73 ± 0.34	0.58 ± 0.01	0.78 ± 0.02	1.94 ± 0.05	0.33 ± 0.01	3.34 ± 0.01
**Tm**	0.11 ± 0.01	0.45 ± 0.01	0.43 ± 0.01	1.53 ± 0.09	0.11 ± 0.01	0.10 ± 0.01	0.29 ± 0.02	0.07 ± 0.01	0.53 ± 0.01
**Yb**	0.76 ± 0.03	2.91 ± 0.03	2.34 ± 0.08	10.3 ± 0.28	0.86 ± 0.04	0.78 ± 0.04	2.00 ± 0.02	0.53 ± 0.03	3.39 ± 0.03
**Lu**	0.12 ± 0.01	0.45 ± 0.01	0.33 ± 0.01	1.63 ± 0.08	0.29 ± 0.01	0.11 ± 0.01	0.32 ± 0.01	0.09 ± 0.01	0.52 ± 0.02
**∑REEY**	37.3	143	133	426	49.3	-	77.6	25.4	172
**∑REE**	30.6	92.0	99.8	326	35.6	18.9	55.2	22.1	137
**Yb_{SN}/Nd_{SN}**	1.72	2.17	1.62	1.79	1.93	3.50	2.37	2.35	1.46
**Dy_{SN}/Nd_{SN}**	1.35	1.78	1.47	1.50	1.30	2.07	1.86	0.79	1.37
**Dy_{SN}/Yb_{SN}**	0.78	0.82	0.91	0.84	0.67	0.59	0.78	0.34	0.94
**$Ce_{SN}/Ce_{SN}*$**	0.72[1]	0.67	0.89[1]	0.59	0.35	1.42	0.58	1.06[1]	0.63
**Gd_{geo}**	1.13	4.10	3.81	15.6	1.12	0.57	2.32	0.34	5.46
**Gd_{AN}**	0.53	-	-	-	3.07	0.29	-	-	-
**Gd_{AN} %**	32	-	-	-	73	33	-	-	-
**$Gd_{SN}/Gd_{SN}*$**	1.45	1.06	1.03	1.03	3.69	1.48	0.96	1.17	0.99

Elements	Antarctica		
	Brazilian Base	North Lake	South Lake
**Y**	160 ± 2.91	126 ± 3.88	200 ± 2.20
**La**	174 ± 5.35	142 ± 4.74	220 ± 4.04
**Ce**	338 ± 0.52	283 ± 5.17	415 ± 08.32
**Pr**	64.8 ± 0.79	50.4 ± 1.49	76.2 ± 0.74
**Nd**	266 ± 3.01	207 ± 4.44	324 ± 0.37
**Sm**	67.7 ± 0.07	52.0 ± 1.53	88.9 ± 2.95
**Eu**	13.9 ± 0.20	10.8 ± 0.18	15.7 ± 0.47
**Gd**	55.0 ± 0.18	40.9 ± 0.79	69.3 ± 3.84
**Tb**	7.94 ± 0.06	5.70 ± 0.16	9.60 ± 0.51
**Dy**	35.9 ± 0.67	24.7 ± 0.79	41.3 ± 1.55
**Ho**	5.70 ± 0.01	4.20 ± 0.14	6.60 ± 0.16
**Er**	16.4 ± 0.10	12.1 ± 0.17	19.4 ± 0.76
**Tm**	1.99 ± 0.05	1.70 ± 0.06	2.33 ± 0.02
**Yb**	12.6 ± 0.30	9.70 ± 0.35	15.0 ± 0.64
**Lu**	1.88 ± 0.06	1.39 ± 0.05	2.12 ± 0.08
**∑REEY**	1222	972	1505
**∑REE**	1062	846	1305
**Yb_{SN}/Nd_{SN}**	0.59	0.58	0.57
**Dy_{SN}/Nd_{SN}**	0.94	0.83	0.89
**Dy_{SN}/Yb_{SN}**	1.61	1.44	1.56
**Ce_{SN}/Ce_{SN}***	0.77	0.81	0.77
**Gd_{geo}**	53.4	39.4	66.9
**Gd_{AN}**	-	-	-
**Gd_{AN} %**	-	-	-
**Gd_{SN}/Gd_{SN}***	1.01	1.02	1.02

[1]Negative anomalies associated with high La concentrations; [2]Data from de Novais et al., 2025 for comparison.

Table S3. Regional and global summary of ∑REE. Gd_{Total}. Gd_{AN}. and Gd anomalies ($Gd_{SN}/Gd_{SN}*$) in tap water and Antarctic water samples. For Gd_{AN}. the minimum value reported corresponds to samples with a Gd/Gd* > 1.25. which is considered indicative of anthropogenic origin

		Europe (n = 29)	North America (n = 15)	South America (n = 7)	Africa (n = 14)	Asia (n = 21)	Oceania (n = 3)	Antarctica (n = 1)	Global (n= 90)
**∑REE ($ng\ L^{-1}$)**	Min.	6.45 (Slovenia - Ljubljana)	9.97 (United States - Woods Hole)	18.1 (Chile - Santiago)	8.39 (Chad - Ndjamena)	12.6 (Nepal – Lalitpu)	18.9 (Australia - Sydney)	846 (North Lake)	6.45 (Slovenia - Ljubljana)
	Max.	174 (France - Corsica)	478 (United States - San Francisco)	414 (Brazil – Rio de Janeiro)	3329 (Nigeria - Benin)	3281 (Indonesia – Tangerang)	326 (Australia - Melbourne)	1305 (South Lake)	3329 (Nigeria - Benin)
	Average	38.6 ± 31.1	73.2 ± 94.5	130 ± 148	664 ± 1074	216 ± 589	90.8 ± 97.0	1071 ± 230	164 ± 484
	Median	31.0	47.2	58.2	107	36.1	55.2	1063	39.5
**Gd_{Total} ($ng\ L^{-1}$)**	Min.	0.35 (Monaco - Monaco)	0.51 (United States - Woods Hole)	0.53 (Chile - Santiago)	0.45 (Chad - Ndjamena)	0.47 (Nepal – Lalitpu)	0.41 (French Polynesia - French Polynesia)	40.9 (North Lake)	0.35 (Monaco– Monaco)
	Max.	61.5 (Germany - Berlin)	29.3 (United States - Washington)	17.5 (Brazil – Rio de Janeiro)	204 (Nigeria - Benin)	144 (Indonesia - Tangerang)	16.3 (Australia - Melbourne)	69.3 (South Lake)	204 (Nigeria - Benin)
	Average	5.10 ± 11.0	4.32 ± 5.53	5.38 ± 4.94	39.6 ± 64.8	10.1 ± 25.4	4.39 ± 4.78	55.0 ± 14.2	10.1 ± 27.3
	Median	2.05	2.30	3.39	5.18	3.17	3.96	55.0	2.79
**Gd_{AN} ($ng\ L^{-1}$)**	Min.	0.37 (Spain - Seville)	0.34 (United States - Woods Hole)	0.31 (Peru - Lima)	0.16 (Reunion Island – Reunion Island)	0.15 (Nepal – Lalitpu)	0.29 (Australia - Sidney)	-	0.15 (Nepal – Lalitpu)
	Max.	61.2 (Germany - Berlin)	27.8 (United States - Washington)	9.32 (Brazil – Rio de Janeiro)	5.60 (Tunisia - Tunis)	6.19 (China – QingDao)	3.07 (Australia - Perth)	-	61.2 (Germany - Berlin)
	Average	5.92 ± 12.5	3.01 ± 6.29	2.65 ± 3.34	2.06 ± 2.03	1.45 ± 1.65	1.29 ± 1.54	-	3.78 ± 8.55
	Median	1.21	1.26	1.62	1.40	0.91	0.53	-	1.24
**Gd/Gd***	Min.	1.25 (France - Corsica; Czech Rep. - Rimov)	1.27 (El Salvador - San Salvador)	1.35 (Peru - Lima)	1.25 (Algeria – Algiers; Reunion Island – Reunion Island)	1.26 (Malaysia – Kuala Lumpur)	1.45 (Australia - Adelaide)	-	1.25 (France - Corsica; Czech Rep. – Rimov; Algeria – Algiers)
	Max.	187 (Germany - Berlin)	20.5 (United States - Washington)	2.53 (Brazil – Brasilia)	6.94 (South Africa - Johannesburg)	7.58 (Japan - Tokio)	3.69 (Australia - Perth)	-	187 (Germany - Berlin)
	Average	11.0 ± 33.6	3.42 ± 4.29	1.82 ± 0.46	2.40 ± 1.91	2.43 ± 1.62	2.21 ± 1.28	-	6.34 ± 21.3
	Median	2.02	1.99	1.75	1.75	2.03	1.48		1.93

Table S4. Speciation results for GdCAs (ng·kg⁻¹) and the percentage of anthropogenic Gd concentration for the samples for which individual GdCAs could be quantified.

Country**	GdCAs		
	Dotarem	Magnevist	Prohance
Barcelona, Spain*	25 ± 3 (83%)	<LOD	<LOD
Zurich, Switzerland*	4.9 ±0.5 (97%)	<LOD	<LOD
Lausanne, Switzerland*	6.0 ± 0.7 (96%)	<LOD	<LOD
Dublin, Ireland*	14 ± 2 (83%)	<LOD	<LOD
Seoul, Korea*	3.3 ± 0.4 (47%)	<LOD	<LOD
Lisbon, Portugal	2.3 ± 0.3 (51%)	<LOD	<LOD
Brasilia, Brazil	5.3 ± 0.6 (73%)	<LOD	<LOD
Wedding/Berlin, Germany	23 ± 3 (46%)	<LOD	<LOD
Zoologischer garten/ Berlin, Germany	46 ± 5 (39%)	<LOD	<LOD
Budapest, Hungary	25 ± 3 (72%)	<LOD	<LOD

*Orani et al.. 2025 (AM Orani. FC Novais. S Azemard. V Hatje. A new HPLC-ICPMS method for Gd speciation in freshwaters using ion exchange chromatography and aqueous mobile phases. Journal of Hazardous materials. 2025. under review); **For speciation analyses. we tested samples with anthropogenic anomalies. However. not all samples showed detectable Gd compounds. likely due to post-collection degradation.

Table S5. Socioeconomic and infrastructure indicators for the countries sampled. including population size. number of magnetic resonance imaging (MRI) units. gross domestic product (GDP) per capita. annual volume of wastewater produced (from domestic. hospital. and industrial sources). and Human Development Index (HDI).

	Countries	Population (2023)[1]	MRI units (2024)[2]	GDP per capita (US$) (2023)[3]	Wastewater million_m3/yr (2020)[4]	HDI (2023)[4]
Europe	Albania	2.745.972	12	8.575.2	27.490.550	0.81
	Austria	9.131.761	241	56.033.6	1.899.227.680	0.93
	Belgium	11.787.423	136	54.700.9	895.506.160	0.95
	Croatia	3.859.686	67	21.865.5	404.530.040	0.89
	Cyprus	1.344.976	18	36.551.4	25.890.000	0.91
	Czech Republic	10.864.042	111	31.591.2	908.025.320	0.92
	Denmark	5.946.952	54	68.453.9	541.705.060	0.96
	Estonia	1.370.286	23	30.133.3	270.934.510	0.89
	France	68.287.487	1229	44.690.9	4.403.033.770	0.92
	Georgia	3.715.483	28	8.283.7	120.573.438	0.84
	Germany	83.280.000	2852	54.343.2	7.511.095.460	0.96
	Greece	10.405.588	389	23.400.7	1.354.988.420	0.91
	Hungary	9562314	56	23.310.0	345.793.220	0.93
	Ireland	5.307.600	87	103.887.8	805.920.270	0.95
	Italy	58.993.475	1939	39.003.3	4.975.361.310	0.92
	Latvia	1.877.445	27	22.502.8	209.373.600	0.89
	Lithuania	2.786.844	35	27.786.0	181.310.150	0.90
	Monaco	38.956	-	256.580.5	7.662.050	-
	Norway	5.519.594	157	87.925.1	899.373.070	0.97
	Poland	37.970.874	486	22.056.7	2.096.694.340	0.91
	Portugal	10.578.174	185	27.331.2	522.144.060	0.89
	Romania	19.059.479	283	18.404.3	1.665.633.370	0.85
	Russia	143.533.851	-	14.889.0	143.533.851	0.83
	Slovenia	2.120.461	36	32.610.1	151.966.260	0.93
	Spain	48.347.910	979	33.509.0	4.284.286.610	0.92
	Switzerland	8.888.093	380	99.564.7	1.434.614.140	0.97
	Ukraine	37.732.836	151	5069.7	2.044.707.000	0.78
	United Kingdom	68.350.000	964	49.463.9	5.978.753.700	0.95
South America	Argentina	45.808.747	650	13.858.2	2.971.941.870	0.87
	Brazil	211.140.729	3225	10.294.9	18.492.493.160	0.79
	Chile	19.658.835	222	17.067.8	1.128.134.310	0.88
	Colombia	52.321.152	350	6.947.4	1.785.623.200	0.79
	Ecuador	17.980.083	20	6.609.8	640.073.690	0.78
	Peru	33.845.617	23	7.906.6	1.019.479.740	0.79
	Uruguay	3.485.152	10	23.906.5	80.779.240	0.86
North America	Antiqua and Barbuda	93.316	2	21.787.1	4.884.733	0.85
	Bahamas	399.440	2	35.896.5	32.075.948	0.82
	Canada	40.097.761	366	53.431.2	7.051.475.650	0.94
	Costa Rica	5.047.561	10	-	369.769.290	0.83
	Cuba	11.019.931	24	9605.3	1.136.426.080	0.76
	El Salvador	6.309.624	7	5.391.1	213.120.190	0.68
	Guatemala	18.124.838	27	5.762.8	1.454.504.480	0.66
	Jamaica	2.839.786	4	6.839.7	99.046.423	0.72
	Mexico	129.739.759	336	13.790.0	7.552.298.900	0.79
	Nicaragua	6.823.613	12	2.612.9	456.391.780	0.71
	Panama	4.458.759	12	18.686.4	249.788.660	0.84
	St. Vicent and the Grenadines	101.323	2	10.520.4	4.078.920	0.80
	Sint Maarten	42.749	-	38.077.5	3.166.786	-
	Trinidad and Tobago	1.367.510	6	20.016.2	256.405.500	0.81
	United States	334.914.895	12234	82.769.4	67.620.412.650	0.94

Cont.	Countries	Population (2023)[1]	MRI units (2024)[2]	GDP per capita (US$) (2023)[3]	Wastewater million_m3/yr (2020)[4]	HDI (2023)[4]
Africa	Algeria	46.164.219	150	5.364.0	805.514.690	0.76
	Chad	19.319.064	0	680.6	63.500.703	0.42
	Ghana	33.787.914	2	2.260.3	525.362.140	0.63
	Kenya	55.339.003	50	1.952.3	224.736.720	0.63
	Mali	23.769.127	4	869.3	124.423.130	0.42
	Nigeria	227.882.945	58	1.596.6	2.289.000.000	0.56
	Reunion Island	892.102	-	-	-	-
	Senegal	18.077.573	-	1.706.4	237.268.450	0.53
	Seychelles	119.773	1	17.879.2	5.985.793	0.85
	South Africa	63.212.384	154	6.022.5	3.670.538.860	0.74
	Togo	9.304.337	1	985.7	31.354.878	0.55
	Tunisia	12.200.431	74	3.977.7	276.016.630	0.75
	Zambia	20.723.965	1	1.330.7	89.308.050	0.59
	Zimbabwe	16.340.822	4	2.156.0	226280470	0.60
Asia	Armenia	2.990.900	12	8.053.0	96.336.106	0.81
	Bahrain	1.577.059	14	29.218.9	136.866.190	0.90
	Bangladesh	171.466.990	70	2.551.0	568.548.000	0.69
	China	1.410.000.000	6685	12.614.1	63.727.700.320	0.80
	India	1.440.000.000	2031	2.480.8	18.627.271.700	0.69
	Indonesia	281.190.067	69	4.876.3	13.844.605.420	0.73
	Iran	90.608.707	303	4.465.6	2.598.515.980	0.80
	Israel	9.756.600	63	52.642.4	575.282.900	0.92
	Japan	124.516.650	6594	33.766.5	12.201.954.380	0.93
	South Korea	51.712.619	1497	33.121.4	7.279.951.460	0.94
	Kuwait	4.853.420	84	33.729.8	290.972.660	0.85
	Malaysia	35.126.298	92	11.379.1	4.354.211.390	0.82
	Nepal	29.694.614	55	1.377.6	261.949.456	0.62
	Pakistan	247.504.495	80	1.365.3	5.672.024.730	0.54
	Qatar	2.656.032	-	80.195.9	276.789.770	0.89
	Saudi Arabia	33.264.292	388	32.094.0	2.034.777.730	0.90
	Singapore	5.917.648	44	84.734.3	552.739.780	0.95
	Syria	23.594.623	45	1.051.7	341.024.140	0.56
	Thailand	71.702.435	63	7.182.0	5.135.763.650	0.80
	Turkey	85.325.965	1490	13.105.7	4.313.014.120	0.85
	Vietnam	100.987.686	51	4717	2.208.997.910	0.77
Oceania	Australia	26.658.948	369	64.820.9	2.142.762.560	0.96
	French Polynesia	281.118	-	20.738.6	19.077.357	-
	New Zealand	5.223.100	65	48.280.8	530.056.710	0.94
	Antarctica	-	No MRI units	-	-	-

[1] World Bank Group. *Countries and Economies*. World Bank Data. 2025https://data.worldbank.org/country/

[2] OECD Data Explorer – Medical technology availability. 2025. https://data-explorer.oecd.org/

[3]Jones et al.. 2021. DOI: https://doi.org/10.5194/essd-13-237-2021. https://essd.copernicus.org/articles/13/237/2021/

[4]UNITED NATIONS DEVELOPMENT PROGRAMME (UNDP). *Human Development Index*. Data Center. Human Development Reports. 2025. <https://hdr.undp.org/data-center/human-development-index

Table S6. Correlation values between Gd_{SN}/Gd_{SN}^* with magnetic resonance imaging machines (MRI number). population. and annual volume of wastewater (WW) generated. Significant values ($p < 0.05$) are presented in bold.

	Europe	North America	South America	Africa	Asia	Oceania*	Global
	Gd_{SN}/Gd_{SN}^*						
MRI number	**0.83**	**1.00**	**0.88**	**0.64**	**0.89**	-	0.24
Population	**0.62**	**0.94**	**0.92**	0.05	0.25	-	0.02
WW million $m^3\ yr^{-1}$	**0.70**	**1.00**	**0.87**	**0.75**	0.54	-	0.12

Where * n is too small to perform correlation.
